# Thorough Characterization of Two Sessein Derivatives with Potential Biological Activity

**DOI:** 10.3390/molecules31020286

**Published:** 2026-01-13

**Authors:** Abraham Gómez-Rivera, Cristian Octavio Barredo-Hernández, Santiago Santos-Vázquez, Carlos Ernesto Lobato-García, Ammy Joana Gallegos-García, Ricardo López-Rodríguez, Laura Alvarez, Ma Dolores Pérez-García, Manasés González-Cortazar, Jorge Luis Torres-López, Eric Jaziel Medrano-Sánchez

**Affiliations:** 1 División Académica de Ciencias Básicas, Universidad Juárez Autónoma de Tabasco, Carretera Cunduacán-Jalpa Km. 0.5, Cunduacán 86690, Tabasco, Mexico; abraham.gomez@ujat.mx (A.G.-R.); cristian_ba99@hotmail.com (C.O.B.-H.); santiagosantsv@gmail.com (S.S.-V.); carlos.lobato@ujat.mx (C.E.L.-G.); ricardo.lopezr@ujat.mx (R.L.-R.); 2 División de Ciencias Básicas e Ingeniería, Universidad Popular de la Chontalpa, Carretera Cárdenas-Huimanguillo Km 2 S/N, Ranchería, Invitab Paso y Playa, Cárdenas 86556, Tabasco, Mexico; joana90102010@gmail.com; 3 Centro de Investigaciones Químicas, Universidad Autónoma del Estado de Morelos, Cuernavaca 62209, Morelos, Mexico; lalvarez@uaem.mx; 4 Centro de Investigación Biomédica del Sur, Instituto Mexicano del Seguro Social, Argentina No. 1, Col. Centro, Xochitepec 62790, Morelos, Mexico; lola_as@yahoo.com.mx (M.D.P.-G.); gmanases2000@gmail.com (M.G.-C.); 5 Laboratorio de Biotecnología, Universidad Politécnica del Centro, Carretera Villahermosa-Teapa Km. 22.5, Tumbulushal, Villahermosa 86290, Tabasco, Mexico; quimb51jl@gmail.com

**Keywords:** *Salvia sessei*, esterified derivatives, structural elucidation, semisynthesis

## Abstract

The diterpene sessein, isolated from *Salvia sessei*, is a metabolite of interest due to its conjugated *p*-quinone system, *δ*-lactone ring, and phenolic hydroxyl in C-12. These functionalities make it an ideal starting point for reactivity studies and semi-synthetic derivatization. In this work, we report the obtainment of two derivatives by selective esterification of phenolic hydroxyl in C-12, through acetylation and benzoylation reactions under mild conditions and with high yields. The structures were characterized by UPLC-MS, FTIR, and NMR spectroscopy ^1^H, ^13^C, and 2D, which allowed to precisely confirm the modifications made in the derivatives. These results confirm that hydroxyl in C-12 constitutes a privileged site of reactivity within the royleanone family, consolidating sessein as a versatile nucleus for the generation of derivatives. Finally, the preliminary evaluation of the antimicrobial activity showed that sessein shows a broad spectrum of action against Gram-positive, Gram-negative, and *Candida albicans* strains. The acetylated derivative showed an increase in activity against gram-negative bacteria, while the benzoyl derivative had a loss of effect at the concentrations evaluated. These findings demonstrate that structural modifications influence the properties of the derivatives with respect to the compound sessein.

## 1. Introduction

Natural products have historically been an essential source of novel chemical structures, the study of which has allowed not only drug discovery, but also the understanding of fundamental phenomena of organic reactivity [1,2]. Among them, the royleanone-type diterpenes, which belong to the abietane family, are of interest due to the presence of a conjugated *p*-quinone skeleton and a phenolic hydroxyl group at position 12 [3], which provides structural versatility and allows for the exploration of structure–reactivity relationships through semi-synthetic transformations [4,5,6].

The literature on royleanones has documented modifications in phenolic and quinone positions, mainly through oxidation, reduction, and esterification reactions [7,8,9,10], as reported by Merecz-Sadowska et al., 2024, where they obtained six esters of the metabolite 7α-acetoxy-6*β*-hydroxyroyleanone isolated from *Plectranthus grandidentatus* [11], as well as the three esterified derivatives of the metabolite 6,7-dehydroroyleanone obtained from the species *Plectranthus aliciae* that were synthesized by Filipe et al., 2023 [12]. Therefore, their structural characteristics not only explain their particular reactivity, but also make them attractive for the study of derivatization reactions [13,14], which has shown that structural changes can alter physicochemical properties such as lipophilicity, polarity, and electronic stability, as well as differences in biological activities between derivatives and their precursors [13,15].

Diterpene sessein lies within the royleanone family and is a metabolite isolated from *Salvia sessei*, which has a *δ*-lactone ring in its structure, and its anti-inflammatory, antioxidant, and antibacterial activity has been reported [16]. Therefore, sessein is of special interest to perform derivatization reactions, as reported by Jiménez et al., 1988, where he mentions the obtaining and partial characterization of the acetylated derivative at position 12 [3] and to our knowledge, this is the only report of an esterified derivative of sessein, this represents an area of opportunity to obtain semi-synthetic derivatives from this diterpene, providing information on the selectivity and behavior of *p*-quinone systems in the face of structural modifications, as well as establishing reactivity patterns, evaluating the influence of substituents on the stability of the compounds and correlating changes structural with modifications in their observable properties.

In this context, the present work aimed at the synthesis of two esterified derivatives of sessein, obtained by acetylation and benzoylation reactions of the hydroxyl group in position 12. The compounds were characterized using spectroscopic techniques (UPLC-MS, FTIR, NMR ^1^H, ^13^C, and 2D experiments), which allowed to accurately confirm their structures, and a preliminary evaluation of their antimicrobial activity was carried out to explore possible correlations between chemical modifications and their biological activity.

## 2. Results and Discussion

### Obtention and Characterization of the Starting Compound and Derivatives

The metabolite (**1**) was obtained as yellow crystals (m.p. 181–182 °C) and with an extraction yield of 1.8% from the dried **SsA** extract. The acetylated derivative (**1a**) (m.p. 194–196 °C) was obtained as a light-yellow powder with 92% reaction yield, and the benzoylate derivative (**1b**) (m.p. 210–212 °C) was obtained as a light-yellow powder with a reaction yield of 95%. The melting points for **1** and **1a** correspond with what was previously reported [3,16].

Compound **1** presented an ion at *m*/*z* 403.18, which corresponds to the molecular weight of sessein with the acquisition of a proton [M + H]^+^, in addition to the fragment at *m*/*z* 343.26 [M − 60]^+^ attributed to the loss of the acetyl group. For **1a**, the ion at *m*/*z* 445.23 [M + H]^+^ matches the molecular weight of the acetylated derivative of sessein with the gain of a proton, and the fragment at *m*/*z* 385.19 [M − 60]^+^ to the loss of an acetyl group. Similarly, the ion at *m*/*z* 343.17 [M − 102]^+^ is consistent with the loss of the acyl and acetyl groups, while the ion at *m*/*z* 315.24 [M − 129]^+^ corresponds to the loss of two acyl groups and the isopropyl group. Finally, the ion at *m*/*z* 297.23 [M − 146]^+^ corresponds to the loss of the acyl, acetyl, and isopropyl groups. The results of **1** and **1a** are consistent with those reported by Jiménez et al., 1988 [3], who present some characteristic fragments of sessein and its acetylated derivative. In the case of compound **1b**, the ion at *m*/*z* 507.48 was observed, which corresponds to the molecular weight of the protonated benzoylated derivative of sessein [M + H]^+^. Likewise, the following peaks were observed within the fragmentation pattern: *m*/*z* 463.40 [M − 44]^+^ due to the loss of CO_2_; *m*/*z* 447.39 [M − 60]^+^ for the loss of the acetyl group; and *m*/*z* 403.49 [M − 104]^+^ for the elimination of the benzoyl group.

The compounds were analyzed by FTIR to determine the functional groups present in their structures. Table 1 presents the assignment of the observed FTIR absorption bands for **1**, **1a**, and **1b**, which are discussed below.

The FTIR spectrum of **1** shows characteristic bands of the hydroxyl group, specifically at 3415 and 1038 cm^−1^ that correspond to the stretches of the O-H and C-O bonds, respectively. For the methyl groups, the absorption bands at 2974 cm^−1^ and 2876 cm^−1^ correspond to the asymmetrical and symmetrical stretches of the C-H bond, respectively; additionally, the band at 1370 cm^−1^ that corresponds to the symmetrical torsion of the C-H bonds is observed. In the case of methylenes, the 2942 cm^−1^ bands for the asymmetrical stretching of the C-H bond and the 1469 cm^−1^ band for the scissor twisting of the H-C-H are observed. In addition, in the case of the ester group, the absorption bands at 1740, 1236, and 1216 cm^−1^ correspond to the stretching of the C=O bond and the asymmetrical and symmetrical stretches of the C-O bonds, respectively. For the isopropyl group, the bands at 1172 and 1146 cm^−1^ are due to the asymmetric stretching of the HC-CH_3_ bonds. Finally, for the quinone group, the 1651 cm^−1^ band corresponds to the C=C bond stretch and the 1635 cm^−1^ band corresponds to the conjugated C=C stretch. The bands observed for **1** agree with those reported in the literature for sessein [3].

In the spectra of **1a** and **1b**, the characteristic band of the O-H stretch at 3415 cm^−1^ was not observed, so the loss of the proton related to the hydroxyl group was confirmed. For **1a** and **1b,** all other bands were similar to those of **1**. The bands observed for **1a** agree with those reported in the literature. For **1b**, two bands (750 and 709 cm^−1^) located in the fingerprint region correspond to the flexing of the hydrogen atoms out of the plane characteristic of a monosubstituted aromatic ring.

Based on the above information, we established that the isolated metabolite (**1**) corresponds to the diterpene sessein, while **1a** and **1b** are its derivatives obtained by esterification. To corroborate this structural assignment, metabolite **1** and derivatives **1a** and **1b** were analyzed by 1D and 2D NMR spectroscopy (Table 2).

**Figure 1:**
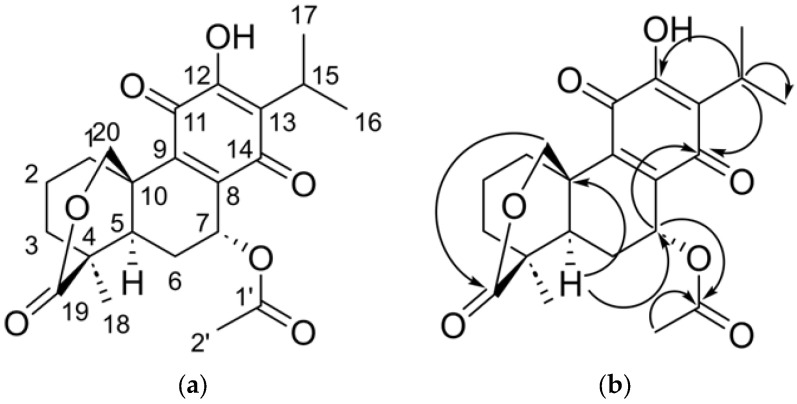
(**a**) The chemical structure and (**b**) principal heteronuclear two- or three-bond correlations (HMBC) of compound **1**.

For compound **1**, two carbonyls conjugated at *δ*_C_ = 183.5 ppm (C-11) and 184.6 ppm (C-14) are identified, as well as signals corresponding to carbons of a quinone group at *δ*_C_ = 150.9 ppm (C-12) and 123.0 ppm (C-13), with the absence of associated aromatic protons. The presence of a phenolic OH signal at *δ*_H_ = 7.02 ppm (s), with HMBCs towards C-11, C-12, and C-13, places this group in the C ring of the abietane-type base structure, confirming the characteristic *p-*benzoquinonic nature of royleanone derivatives (Figure 1).

The typical pattern of an isopropyl group with methine H-15 at *δ*_H_ = 3.21 ppm (hept, *J* = 7.4 Hz) correlated in HSQC with *δ*_C_ = 24.4 ppm, and its methyl groups H-16 and H-17 at *δ*_H_ = 1.19 ppm and 1.23 ppm (d, *J* = 7.4 Hz) correlated with *δ*_C_ = 19.73 ppm and 19.91 ppm, respectively. The HMBC of H-15 to C-12, C-13, and C-14 confirm its anchoring to the quinone ring. Likewise, a signal at *δ*_H_ = 6.01 ppm (dd, *J* = 1.9, 3.8 Hz) is observed, correlated with *δ*_C_ = 62.1 ppm. The HMBC of H-7 to the carbonyl *δ*_C_ = 169.2 ppm (C-1′) confirms the presence of an acetate group at this position, which explains the shift to high frequencies of the proton. In addition, the data show equatorial–axial (*J* = 3.8 Hz) and equatorial–equatorial (*J* = 1.9 Hz) couplings with the protons of C-6, confirming the *β*-equatorial orientation of H-7, which is the base proton of the acetate group as previously reported [3]. In the case of the lactonic ring, the diastereotopic protons of C-20 are observed in *δ*_H_ = 4.86 ppm (d, *J* = 12.3 Hz) corresponding to H-20_pro-*R*_ and 4.27 ppm (dd, *J* = 12.3, 2.1 Hz) for H-20_pro-*S*_, in both the coupling constants corresponding to twin protons (*J* = 12.3 Hz) are observed, in addition, in H-20_pro-S_ the coupling of type W with H-1a (*J* = 2.1 Hz) is observed. Both protons are correlated in HSQC with *δ*_C_ = 73.5 ppm; this shift to high frequencies is produced by a C-O bond in C-20. In the HMBC analysis, these protons show correlations with C-1, C-5, C-9, C-10, and the carbonyl *δ*_C_ = 174.8 ppm (C-19), which establishes the proximity of this oxygenated group to the carbonyl, in accordance with the structure of sessein. The analysis of aliphatic carbons allowed the assignment of the decalin nucleus characteristic of the Abietanes. The system includes signals in C-1 (*δ*_C_ = 35.5 ppm) with its protons *δ*_Ha_ = 1.50 ppm (tdd, *J* = 13.1, 5.3, 2.1 Hz) and *δ*_He_ = 2.86 ppm (d, *br*, *J* = 13.1 Hz), C-2 (*δ*_C_ = 20.9 ppm), and both protons in *δ*_H_ = 1.96–1.84 ppm (m), C-3 (*δ*_C_ = 40.2 ppm) with the protons *δ*_Ha_ = 2.00 ppm (ddt, *J* = 13.3, 5.3, 1.9 Hz) and *δ*_He_ = 1.55 ppm (td, *J* = 13.3, 5.3 Hz). Carbons C-4 (*δ*_C_ = 42.0 ppm), C-5 (*δ*_C_ = 42.7 ppm) with the proton *δ*_H_ = 1.89 ppm (dd, *J =* 14.4, 3.8 Hz) and C-6 (*δ*_C_ = 25.9 ppm) with their two protons *δ*_Ha_ = 1.53 ppm (td, *J* = 14.4, 3.8 Hz) and *δ*_He_ = 2.20 ppm (dt, *J =* 14.4, 3.8 Hz) complete this fused system. In this system, couplings between geminal and axial–axial protons (*J* = 13–14 Hz) are observed, as well as couplings between axial–equatorial and equatorial–equatorial protons (*J* = 3–6 Hz), and finally, the ^4^J_HH_ coupling of type W between the axial–axial protons of H-1 and H-3 is evidenced.

In addition, the tertiary methyl at C-18 is observed as a singlet at *δ*_H_ = 1.23 ppm (*δ*_C_ = 23.2 ppm), with HMBC towards C-3, C-4, C-5, and C-19, confirming its location in the bridge of the A ring. The key HMBC, in particular H-7 → C-1′, H-15 → C-12–C-14, H-20 → C-19, and H-18 → C-3–C-5 and C-19, allow the arrangement of the functional groups to be unambiguously established. The dataset with its characteristic abietane nucleus with a decalin forming the A and B rings, as well as the p-quinone system, the isopropyl group and the hydroxyl in the C ring, the acetate group in C-7, and the C-20–O–C-19 *δ*-lactonic system confirms that compound **1** is the so-called 7α-acetyl-12-hydroxyabieta-8, 13-dien-11,14-dione-19,20-*δ*-lactone, i.e., sessein.

Table 3 presents the spectroscopic data for **1a**, which is discussed below.

**Figure 2:**
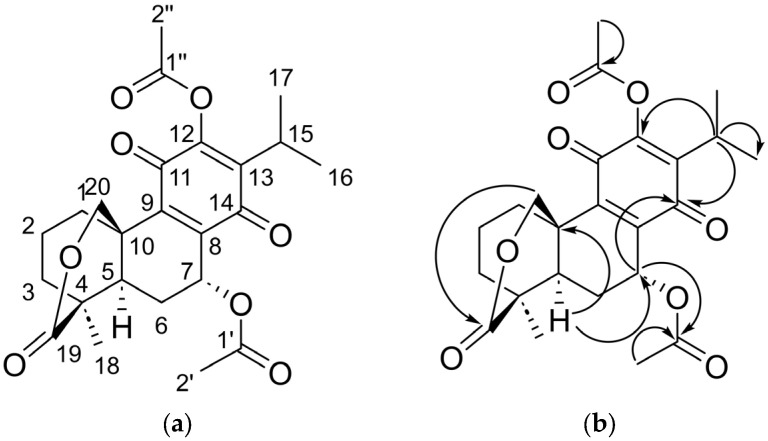
(**a**) The chemical structure and (**b**) principal heteronuclear two- or three-bond correlations (HMBC) of compound **1a**.

The signals obtained by the analysis of ^1^H of **1a** coincide with what was reported by Jiménez et al. in 1988 [3], where a singlet is observed with a *δ*_H_ = 2.34 ppm that integrates for three protons, so it is inferred that they are the methyl protons of the acetyl group introduced at position 12. In addition, in the analysis of ^13^C in comparison to the starting compound, two new signals are observed with *δ*_C_ = 168.3 ppm and *δ*_C_ = 20.4 ppm that are consistent in their assignment to carbons 1″ and 2″, respectively. The remaining signals were like those of the starting compound in both displacement and multiplicity. Therefore, the obtainment of 7α-acetyl-12-O-acetylabieta-8,13-dien-11,14-dione-19,20-*δ*-lactone is confirmed (Figure 2).

Table 4 presents the spectroscopic data for **1b**, which is discussed below.

**Figure 3:**
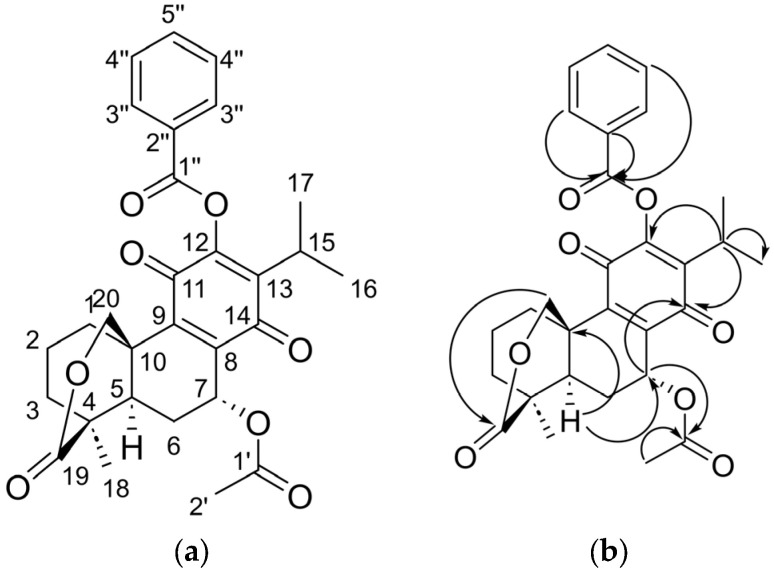
(**a**) The chemical structure and (**b**) principal heteronuclear two- or three-bond correlations (HMBC) of compound **1b**.

The signals obtained by the analysis of ^1^H of **1b** mostly coincide with the signals reported by García et al., 2020 for 6,7-dehydro-12-O-benzoyroyleone [17]. Thus, for **1b**, three proton signals characteristic of a monosubstituted aromatic ring with *δ*_H_ = 8.07 (dd, *J =* 7.8, 1.3 Hz), *δ*_H_ = 7.48 (t, *J =* 7.8 Hz) and *δ*_H_ = 7.63 (tt, *J =* 7.8, 1.3 Hz) were observed, corresponding to the protons in positions *ortho*, *para*, and *meta* with respect to substitution, respectively. These signals were correlated by HMBC to the carbon of a carbonyl belonging to an ester (*δ*_C_ = 163.6 ppm), as well as to the quaternary carbon of the monosubstituted aromatic ring (*δ*_C_ = 128.6 ppm), which are signals absent in the starting compound. Therefore, the obtainment of 7α-acetyl-12-O-benzoylabiate-8,13-dien-11,14-dione-19,20-*δ*-lactone is confirmed (Figure 3).

## 3. Preliminary Antimicrobial Activity

The antimicrobial activity results are presented in Table 5, where it is observed that both **1** and **1a** showed significant antimicrobial activity against Gram-positive and Gram-negative bacteria and the fungus evaluated; metabolite **1** showed activity against five microorganisms, all with MIC **25** μg/mL, of which three were Gram-positive, one Gram-negative, as well as against *C. albicans*. Compound **1a** showed activity against six microorganisms with MIC between **25** and **50** μg/mL, of which two were Gram-positive, four Gram-negative, and against *C. albicans*. **1b** showed no activity against any strain at the concentrations evaluated.

In the case of compound **1**, these results confirm its antimicrobial activity against Gram-positive and Gram-negative bacteria [16]; furthermore, this study reports for the first time the activity of **1** against a fungus (*C. albicans*). Likewise, this is the first report of the antimicrobial activity of **1a** and **1b**, where the presence of the acetyl group in position 12 generated a greater antimicrobial effect against Gram-positive and Gram-negative strains, as well as against *C. albicans*; while the benzoyl group in position 12 showed no activity at the concentrations evaluated. It should be noted that these preliminary results are promising because, although a limited range of concentrations was used, the MICs achieved in this study for **1** and **1a** allow them to be considered as candidates of interest for evaluation against microorganisms relevant to health.

## 4. Materials and Methods

### 4.1. Isolation of the Starting Compound

#### 4.1.1. Obtaining Organic Extract

The collection of *Salvia sessei* Benth. (4 kg) was carried out in the town of San Andrés de la Cal, located within the municipality of Tepoztlán, Morelos, Mexico (18°57′32.684″ N, 99°7′1.639″ W) in November 2023. A specimen previously collected in the same coordinates was deposited in the HUMO-CIByC Herbarium of the Autonomous University of the State of Morelos for safekeeping and taxonomic identification (voucher 33909).

The plant material was dried in a furnace (Riossa H-33, VelaQuin, Jalisco, Mexico) and pulverized in a mill (Pulvex MP300, Mexico City, Mexico). The dried plant material was macerated with *n*-hexane (Merck, Darmstadt, Germany) for 24 h, which was filtered and concentrated by rotation evaporation (Büchi R-100, Flawil, Switzerland) until the hexanic extract (**SsH**) was obtained. This process was repeated twice to perform an exhaustive extraction of the metabolites with this solvent. Subsequently, the residual dry plant material was macerated with acetone (Merck, Darmstadt, Germany) following the same methodology until the acetone extract (**SsA**) was obtained.

From the maceration of 1 kg of dried and ground plant material, the following extracts were obtained in quantities and yields: **SsH** (11.5 g, 1.15%); **SsA** (41.5. g, 4.15%).

#### 4.1.2. Chromatographic Fractionation and Isolation of Sessein (**1**)

The **SsA** extract (30 g) was adsorbed on silica gel (Silica Gel 60, Merck, Darmstadt, Germany) and placed in a glass column (8 cm × 30 cm) with silica gel (300 g) as the stationary phase. To elute the column as a mobile phase, an n-hexane/ethyl acetate gradient (Merck, Darmstadt, Germany) with a polarity increase of 5% *v*/*v* was used, collecting 60 fractions of 500 mL each. The fractions were concentrated in a rotary evaporator (Büchi R-100, Flawil, Switzerland) at reduced pressure and analyzed by normal-phase thin-layer chromatography (TLC) (silica gel at 60 F254; Merck, Darmstadt, Germany). The fractions that revealed similarity in their chemical content were grouped into 10 fractions (**SsA**1-8). The fraction **SsA-5** showed the presence of a yellow solid was observed, similar to what was previously reported for sessein (**1**) [16]; therefore, it was subjected to chromatographic fractionation in column in a silica gel column (150 g) and eluted with an *n*-hexane/ethyl acetate gradient system with an increase in polarity of 5%, generating 50 fractions, which were analyzed by TLC in normal phase and grouped into 8 subfractions (**SsA**-5R1 to **SsA**-5R8). Subfractions 3–5 showed a yellow solid so they were grouped (0.9 g) and adsorbed with reversed-phase silica gel (RP) (0.9 g, RP-18. 40–63 μm; Merck, Darmstadt, Germany) and mounted on a “flash” RP column (Supelco LC18™, Merck, Darmstadt, Germany), using a water/acetonitrile gradient system as a mobile phase, with a polarity change of 5% and collection volumes of 10 mL, obtaining 55 fractions that were concentrated and analyzed by RP TLC (silica gel RP-18 F_254s_; Merck, Darmstadt, Germany). Yellow crystals (0.55 g) were obtained from fraction 10–25, which were analyzed by spectroscopic techniques and identified as sessein (**1**).

### 4.2. Semisynthesis of Derivatives

The derivative 12-O-acetylated (**1a**) was obtained following the methodology reported by Jiménez et al., 1988 [3]: **1** (100 mg, 0.012 mmol) was mixed in acetic anhydride (2 mL; Merck, Darmstadt, Germany) and pyridine (1 mL; Merck, Darmstadt, Germany) and stirred at room temperature for 30 min, and monitored by TLC until the total consumption of **1**.

The derivative 12-O-benzoylate (**1b**) was obtained following the methodology reported by García et al., 2020 [17]: **1** (100 mg, 0.012 mmol) was reacted in pyridine (12 eq.) and benzoyl chloride (12 eq.; Merck, Darmstadt, Germany), stirred for 30 min at room temperature, and monitored by TLC until the total consumption of **1**.

For all compounds, uncorrected melting points were obtained on a melting point apparatus STUART SMP 10 (Bibby Scientific, Staffordshire, UK).

### 4.3. Characterization of the Compounds

#### 4.3.1. UPLC-MS

The molecular weights of the **1**, **1a**, and **1b** were determined using an Acquity UPLC kit (Waters Corp., Milford, MA, USA). The separation system included a quaternary pump, a column furnace, and an autosampler with a photodiode array detector coupled to a Xevo triple quadrupole mass spectrometer at 150 °C. The solvation temperature was 500 °C, and the solvation gas flow rate was 700 L/h of nitrogen. Argon was withdrawn as the impingement gas at a flow rate of 0.10 mL/min (Thermo Fisher Scientific, Bremen, Germany). The compounds (5 μL) were analyzed on an Acquity UPLC BEH RP-18 column (2.1 mm × 50.0 mm, 1.7 μm Water Corp.) with a mobile phase gradient of high purity: (A) water with TFA (0.5%, Sigma-Aldrich, Steinheim, Germany) and (B) acetonitrile of high purity (Merck, Darmstadt, Germany) were eluted at flow rate of 0.3 mL/min. The entire experiment ran for 20 min.

#### 4.3.2. FTIR

The functional groups of the starting compound and derivatives were analyzed according to the methodology reported by Torres-López et al., 2025 [18] by Fourier transform infrared spectroscopy (FTIR) (Nicolet iS50; Thermo Fisher Scientific, Waltham, MA, USA) equipped with an attenuated total reflectance module and a diamond crystal. Spectra were collected in a range of 4000 to 400 cm^−1^ with 32 scans at a set resolution. Subsequently, the spectra obtained were analyzed, identifying the characteristic absorption bands corresponding to the functional groups present, based on data reported in the literature [19].

#### 4.3.3. NMR

The structures of the starting compound and derivatives were identified by ^1^H, ^13^C, and 2D (COSY, HSQC, and HMBC) NMR following the methodology reported by Gómez-Rivera et al., 2018 [16] using the JEOL ECZ 600 MHz NMR spectrometer (JEOL, Tokyo, Japan): for all compounds, the solvent was CDCl_3_ (Sigma-Aldrich, St. Louis, MO, USA). Chemical displacements (*δ*) are expressed in parts per million (ppm) relative to tetramethylsilane (TMS) or residual solvent peaks used as internal references. The following abbreviations were used to describe the signal patterns: s (singlet), d (doublet), t (triplet), q (quartet), m (multiple), etc. The coupling constants (*J*) are reported in hertz (Hz).

### 4.4. Antimicrobial Activity of Compounds

In order to explore possible correlations between chemical modifications and their biological activity, the antimicrobial activity of **1**, **1a**, and **1b** was evaluated following what was reported by Gallegos-García et al., 2022 [20] against the strains: **Sa1**: *Staphylococcus aureus* ATCC 29213, **Sa2**: *Staphylococcus aureus* methicillin-resistant ATCC 43300, **Se1**: *Staphylococcus epidermidis* ATCC 35984, **Se2**: *Staphylococcus epidermidis* ATCC 12228, **Se3**: *Staphylococcus epidermidis* ATCC 1042, **Sh**: *Staphylococcus haemolyticus* (clinical isolate) ATCC 1038. **Ef**: *Enterococcus faecalis* ATCC 29212, **Kp**: *Klebsiella pneumoniae* ATCC 700603, **Pa**: *Pseudomonas aeruginosa* ATCC 27853, **Ec**: *Escherichia coli* ATCC 1042, **Sd**: *Salmonella dublin* ATCC 9676, **Ecl**: *Enterobacter cloacae* ATCC 700323, **Ab**: *Acinetobacter baumannii* ATCC 9736, **Ca**: *Candida albicans* ATCC 10231.

The strains were reseeded on antibiotic agar No. 1 (Bioxon, Becton Dickinson of Mexico, Mexico City, State of Mexico, Mexico) for 24 h at 37 °C.

Cultures with 24 h incubation (37 °C) were used for the assays, and about 3–4 colonies of each strain were taken and diluted in Müeller–Hinton broth (MH; Bioxon, Toluca, Mexico). Inoculums were adjusted using the MacFarland scale of 0.5 (1.5 × 10^8^ CFU/mL). Subsequently, a dilution with distilled water was carried out to obtain 1 × 10^4^ CFU/mL.

The MIC of the extracts was determined by the broth microdilution method. Briefly, the samples (50 mg/mL) were dissolved in a DMSO/water mixture (20:80), and the concentrations tested were 25, 50, 100, and 200 μg/mL. After dilution into the assay wells containing Müeller–Hinton broth and bacterial inoculum, the final DMSO concentration in each well was below 1% (*v*/*v*), a level considered non-inhibitory for microbial growth [21]. Samples were added to sterile 96-well microplates, along with 200 μL MH and 2 μL inoculum (1 × 10^4^ CFU/mL). The viability controls used were MH/DMSO/inoculum and MH/inoculum; Gentamicin (**C+**, 100 μg/mL; Sigma Aldrich, Toluca, Mexico) was used as the reference antibiotic. The plates were incubated at 37 °C for 24 h, and after incubation the MIC was determined by adding 30 μL of a solution (0.05%) of 3-(4,5-dimethylthiazol-2-yl)-2,5-diphenyltetrazolium bromide (MTT, Sigma-Aldrich, Hong Kong, China) in each well, of which purple development was observed if there was viability of bacteria and colorless if there was none. All MIC determinations were performed in three independent replicates within the same assay.

## 5. Conclusions

Two derivatives were semi-synthesized by selective esterification reactions in the phenolic hydroxyl group of sessein (**1**): the acetylated ester (**1a**) and the benzoylated ester (**1b**), which were obtained with excellent yields (>90%), a preliminary analysis of the mass fragmentation patterns and the detailed spectroscopic analysis (FTIR, NMR ^1^H, ^13^C, and two-dimensional experiments) allowed to accurately assign the structures of the compounds obtained. The chemical displacements and correlations observed show the modifications introduced in the electronic environment and corroborate the selectivity of the reaction. These results confirm the hydroxyl group of the *p*-quinone ring in sessein as a relevant reactive site and underline the value of semisynthesis as a tool to expand the structural and exploratory knowledge of natural metabolites.

Finally, the preliminary evaluation of antimicrobial activity shows that these structural modifications impact biological behavior, which reinforces the relevance of studying these reactions as a model to generate new structures that allow a greater understanding of the effects of chemical changes and their relationship with biological properties.

## Figures and Tables

**Table 1:** IR bands observed for compound **1** and derivatives **1a** and **1b**.

Compound	Observed Bands (cm^−1^)
**1**	3415, 2974, 2942, 2876, 1740, 1651, 1635, 1469, 1370, 1236, 1216, 1172, 1146, 1038
**1a**	2972, 2934, 2880, 1737, 1660, 1621, 1451, 1371, 1276, 1213, 1126, 1188, 1173, 1024
**1b**	2972, 2934, 2880, 1737, 1660, 1621, 1451, 1371, 1276, 1213, 1126, 1188, 1173, 1024

**Table 2:** NMR spectroscopic data for compound **1** (^1^H = 600 MHz, ^13^C = 150 MHz, CDCl_3_, ambient temperature, *δ* = ppm, *J* = Hz).

C Position	HSQC		HMBC (H → C)
	*δ*_H_	*δ*_C_	
1	H_a_ 1.50 (tdd, *J =* 13.1, 5.3, 2.1) H_e_ 2.86 (d, *br*, *J* = 13.1)	35.5	2, 3, 5, 10, 20
2	2H 1.96–1.84 (m)	20.9	1, 3, 4, 10
3	H_a_ 2.00 (ddt, *J =* 13.3, 5.3, 1.9) H_e_ 1.55 (td, *J* = 13.3, 5.3)	40.2	1, 2, 4, 5, 18, 19
4	-	42.0	
5	H 1.89 (dd, *J* = 14.4, 2.8)	42.7	3, 6, 7, 9, 10, 18, 19, 20
6	H_a_ 1.53 (dt, *J* = 14.4, 3.8) H_e_ 2.20 (dt, *J =* 14.4, 3.8)	25.9	4, 5, 7, 8, 10
7	H 6.01 (dd, *J =* 1.9, 3.8)	62.1	5, 6, 8, 9, 14, 1′
8	-	140.5	
9	-	143.8	
10	-	37.7	
11	-	183.5	
12	-	150.9	
13	-	123.0	
14	-	184.6	
15	H 3.21 (hept, *J* = 7.4)	24.4	12, 13, 14, 16, 17
16	H 1.19 (d, *J* = 7.4)	19.7	13, 15, 16
17	H 1.23 (d, *J* = 7.4)	19.9	13, 15, 17
18	H 1.23 (s)	23.2	3, 4, 5, 19
19	-	174.8	
20	H_pro-*R*_ 4.86 (d, *J* = 12.3) H_pro-*S*_ 4.27 (dd, *J* = 2.1, 12.3)	73.5	1, 5, 9, 10, 19
1′	-	169.2	
2′	2.07 (s)	21.1	1′
OH	7.02 (s)	-	11, 12, 13

**Table 3:** NMR spectroscopic data for compound **1a** (^1^H = 600 MHz, ^13^C = 150 MHz, CDCl_3_, ambient temperature, *δ* = ppm, *J* = Hz).

C Position	HSQC		HMBC (H → C)
	*δ*_H_	*δ*_C_	
1	H_a_ 1.45 (tdd, *J* = 13.1, 6.3, 2.0) H_e_ 2.65 (d, *J* = 13.1)	35.3	2, 5, 10, 20
2	2H 1.89–1.82 (m)	20.9	1, 3, 4, 10
3	H_a_ 1.99 (ddd, *J* = 11.6, 4.1, 2.0) H_e_ 1.59–1.50 (m)	40.2	1, 2, 4, 5, 19
4	-	42.0	
5	H 1.89–1.82 (m)	42.6	4, 6, 7, 10, 19, 20
6	H_a_ 1.59–1.50 (m) H_e_ 2.18 (dt, *J =* 14.8, 3.9)	26.0	4, 5, 7, 8, 10
7	H 6.00 (dd, *J* = 3.9, 1.8)	62.0	5, 6, 8, 9, 14, 1′
8	-	138.7	
9	-	147.3	
10	-	38.0	
11	-	180.5	
12	-	149.3	
13	-	140.3	
14	-	184.6	
15	H 3.12 (hept, *J =* 7.3)	25.4	12, 13, 14, 16, 17
16	3H 1.21 (d, *J =* 7.3)	20.3	13, 15, 17
17	3H 1.19 (d, *J =* 7.3)	20.2	13, 15, 16
18	3H 1.23 (s)	23.2	3, 4, 5, 19
19	-	174.8	
20	H_pro-*R*_ 4.91 (d, *J =* 12.4) H_pro-*S*_ 4.27 (dd, *J =* 12.4, 2.1)	73.7	1, 9, 10, 19
1′	-	169.3	
2′	3H 2.08 (s)	21.1	1′
1″	-	168.3	
2″	3H 2.35 (s)	20.4	1″

**Table 4:** NMR spectroscopic data for compound **1b** (^1^H = 600 MHz, ^13^C = 150 MHz, CDCl_3_, ambient temperature, *δ* = ppm, *J* = Hz).

C Position	HSQC		HMBC (H → C)
	*δ*_H_	*δ*_C_	
1	H_a_ 1.49 (ddd, *J =* 12.9, 6.3, 2.0) H_e_ 2.59 (d, *br J* = 12.9)	35.4	2, 3, 5, 10, 20
2	H_a_ 1.89–1.74 (m) H_e_ 1.97–1.89 (m)	20.8	1, 3, 4, 10
3	H_a_ 1.99 (d, *br J =* 13.2) H_e_ 1.57–1.42 (m)	40.2	1, 2, 4, 5, 18, 19
4	-	42.0	
5	H 1.81 (dd, *J* = 13.7, 3.8)	42.6	3, 6, 7, 9, 10, 18, 19, 20
6	H_a_ 1.57–1.42 (m) H_e_ 2.13 (dt, *J =* 13.7, 3.8)	26.1	4, 5, 7, 8, 10
7	H 5.94 (dd, *J =* 3.8, 1.8)	62.1	5, 6, 8, 9, 14, 1′
8	-	138.6	
9	-	147.6	
10	-	38.1	
11	-	180.5	
12	-	149.6	
13	-	140.4	
14	-	184.6	
15	H 3.06 (hept, *J =* 7.4)	25.4	12, 13, 14, 16, 17
16	3H 1.14 (d, *J =* 7.4)	20.5	13, 15, 16
17	3H 1.13 (d, *J =* 7.4)	20.2	13, 15, 17
18	3H 1.27 (s)	23.2	3, 4, 5, 19
19	-	174.8	
20	H_pro-*R*_ 4.84 (d, *J =* 12.4) H_pro-*S*_ 4.19 (dd, *J =* 12.4, 2.1)	73.7	1, 5, 9, 10, 19
1′	-	169.3	
2′	3H 2.01 (s)	21.2	1′
1″	-	164.0	
2″	-	128.6	
3″	2H 8.07 (dd, *J =* 7.8, 1.3)	130.6	1″, 2″, 4″, 5″
4″	2H 7.48 (t, *J =* 7.8)	129.0	2″, 3″, 5″
5″	H 7.63 (tt, *J =* 7.8, 1.3)	133.7	3″, 4″

**Table 5:** Antimicrobial activity (MIC μg/mL) of sessein and its esterified derivatives.

Sample	Gram-Positive							Gram-Negative						Fungi
	Sa1	Sa2	Se1	Se2	Se3	Sh	Ef	Kp	Pa	Ec	Sd	Ecl	Ab	Ca
**1**	**25**	**25**	200	**25**	200	200	200	200	**25**	200	200	200	200	**25**
**1a**	200	200	**25**	200	200	200	**25**	**50**	**25**	200	**25**	**25**	200	**25**
**1b**	200	200	200	200	200	200	200	200	200	200	200	200	200	200
**C1**	*	*	*	*	*	*	*	*	*	*	*	*	*	*
**C2**	*	*	*	*	*	*	*	*	*	*	*	*	*	*
**C+**	---	---	---	---	---	---	---	---	---	---	---	---	---	---

**Sa1**: *Staphylococcus aureus* ATCC 29213, **Sa2**: *Staphylococcus aureus* methicillin-resistant ATCC 43300, **Se1**: *Staphylococcus epidermidis* ATCC 35984, **Se2**: *Staphylococcus epidermidis* ATCC 12228, **Se3**: *Staphylococcus epidermidis* ATCC 1042, **Sh**: *Staphylococcus haemolyticus* (Clinical isolate) ATCC 1038. **Ef**: *Enterococcus faecalis* ATCC 29212, **Kp**: *Klebsiella pneumoniae* ATCC 700603, **Pa**: *Pseudomonas aeruginosa* ATCC 27853, **Ec**: *Escherichia coli* ATCC 1042, **Sd**: *Salmonella dublin* ATCC 9676, **Ecl**: *Enterobacter cloacae* ATCC 700323, **Ab**: *Acinetobacter baumannil* ATCC 9736, **Ca**: *Candida albicans* ATCC 10231; **C1** y **C2**: controls of viability (*: bacterial growth); **C+**: positive control (gentamicin 100 μg/mL; ---: No bacterial growth).

## Data Availability

The original contributions presented in this study are included in the article and Supplementary Materials. Further inquiries can be directed to the corresponding author.

## Supplementary Materials

The following supporting information can be downloaded at: https://www.mdpi.com/article/10.3390/molecules31020286/s1.
