# Supplementary material for: Thorough Characterization of Two Sessein Derivatives with Potential Biological Activity
Source: Molecules. 2026 Jan 13;31(2):286. doi: 10.3390/molecules31020286 (PMC12844448; doi:10.3390/molecules31020286)
Supplement: Supplementary file 1 [file molecules-31-00286-s001.zip › molecules-4078725-supplementary.pdf]

Supplementary

Thorough Characterization of Two Sessein Derivatives with Potential Biological Activity

Abraham Gómez-Rivera [1], Cristian Octavio Barredo-Hernández [1], Santiago Santos-Vázquez [1], Carlos Ernesto Lobato-García [1], Ammy Joana Gallegos-García [2], Ricardo López-Rodríguez [1], Laura Alvarez [3], Ma Dolores Pérez-García [4], Manasés González-Cortazar [4], Jorge Luis Torres-López [5] and Eric Jaziel Medrano-Sánchez [1,*]

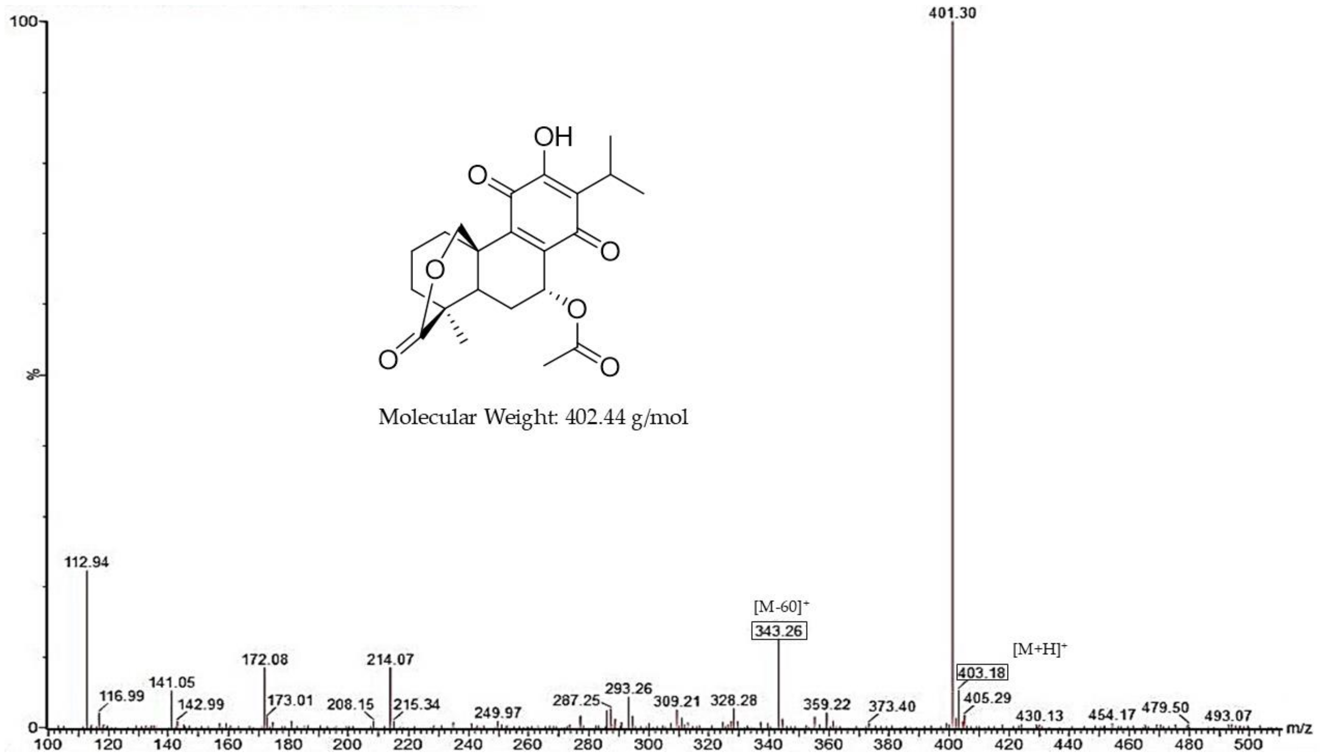

Figure S1. Mass spectrometry of sessein (**1**), ion *m*/*z* 403.18 [M+H]+.

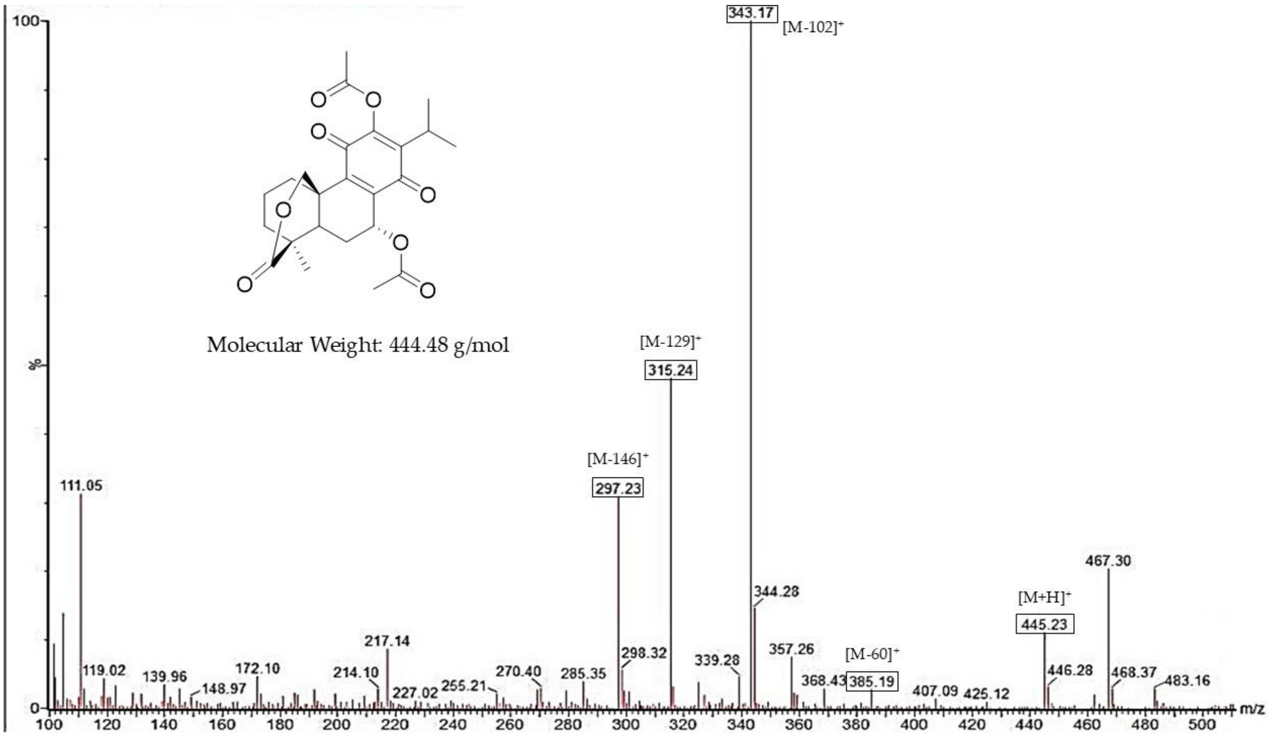

Figure S2. Mass spectrometry of the acetylated derivative (**1a**), ion *m/z* 445.23 $[M+H]^+$.

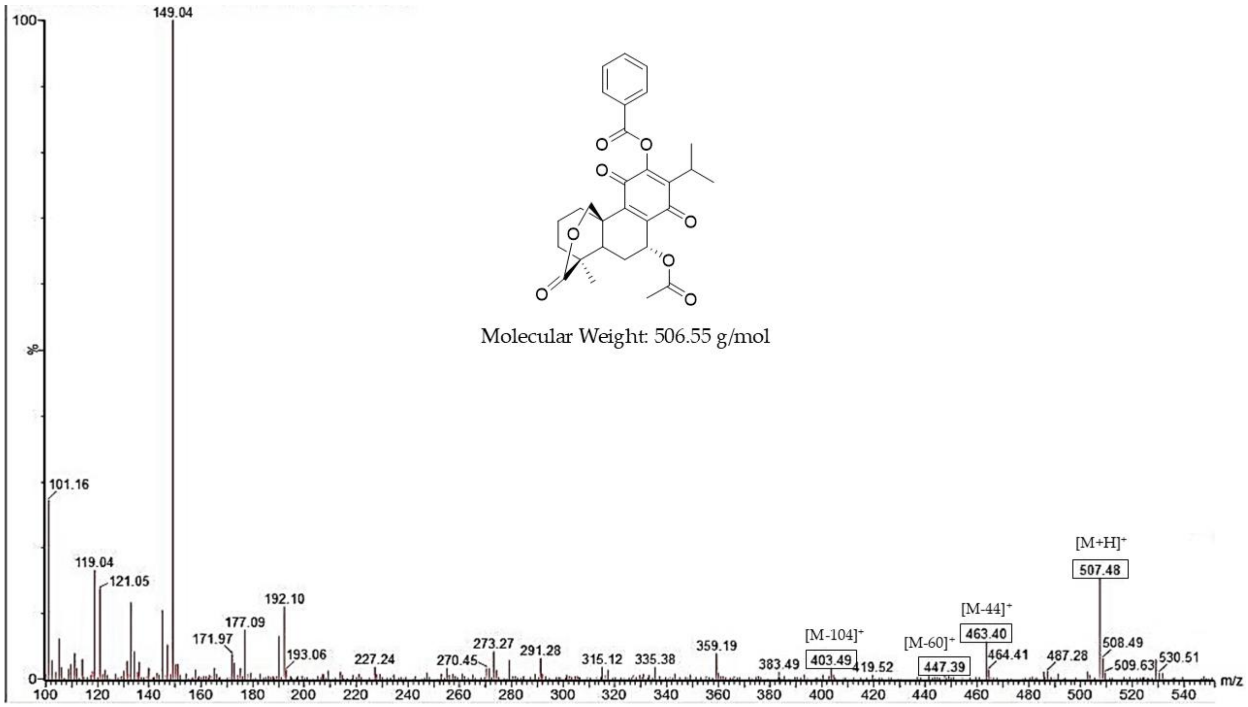

Figure S3. Mass spectrometry of the benzoylated derivative (**1b**), ion *m/z* 507.48 $[M+H]^+$.

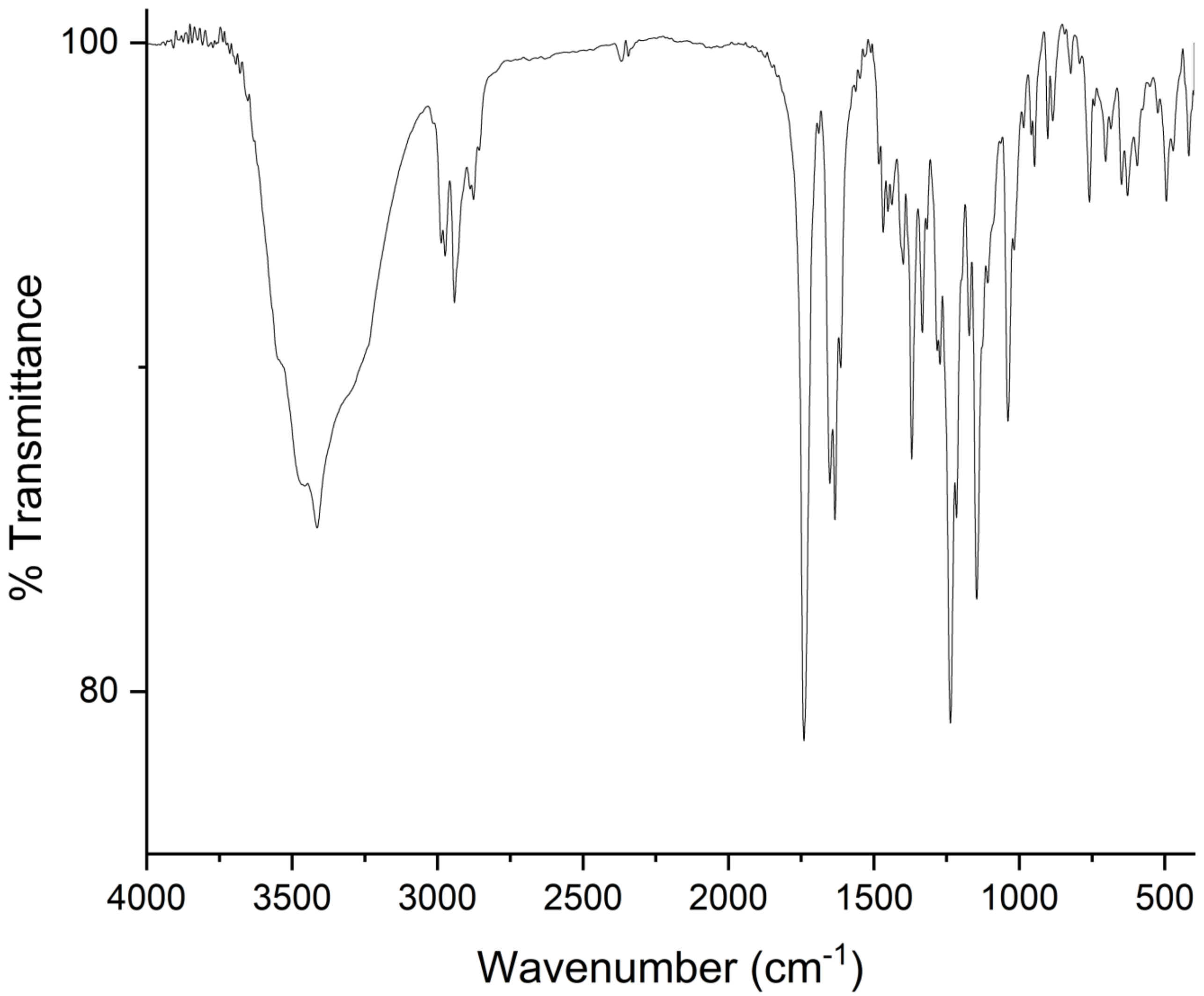

Figure S4.- FTIR spectrum of sessein (**1**)

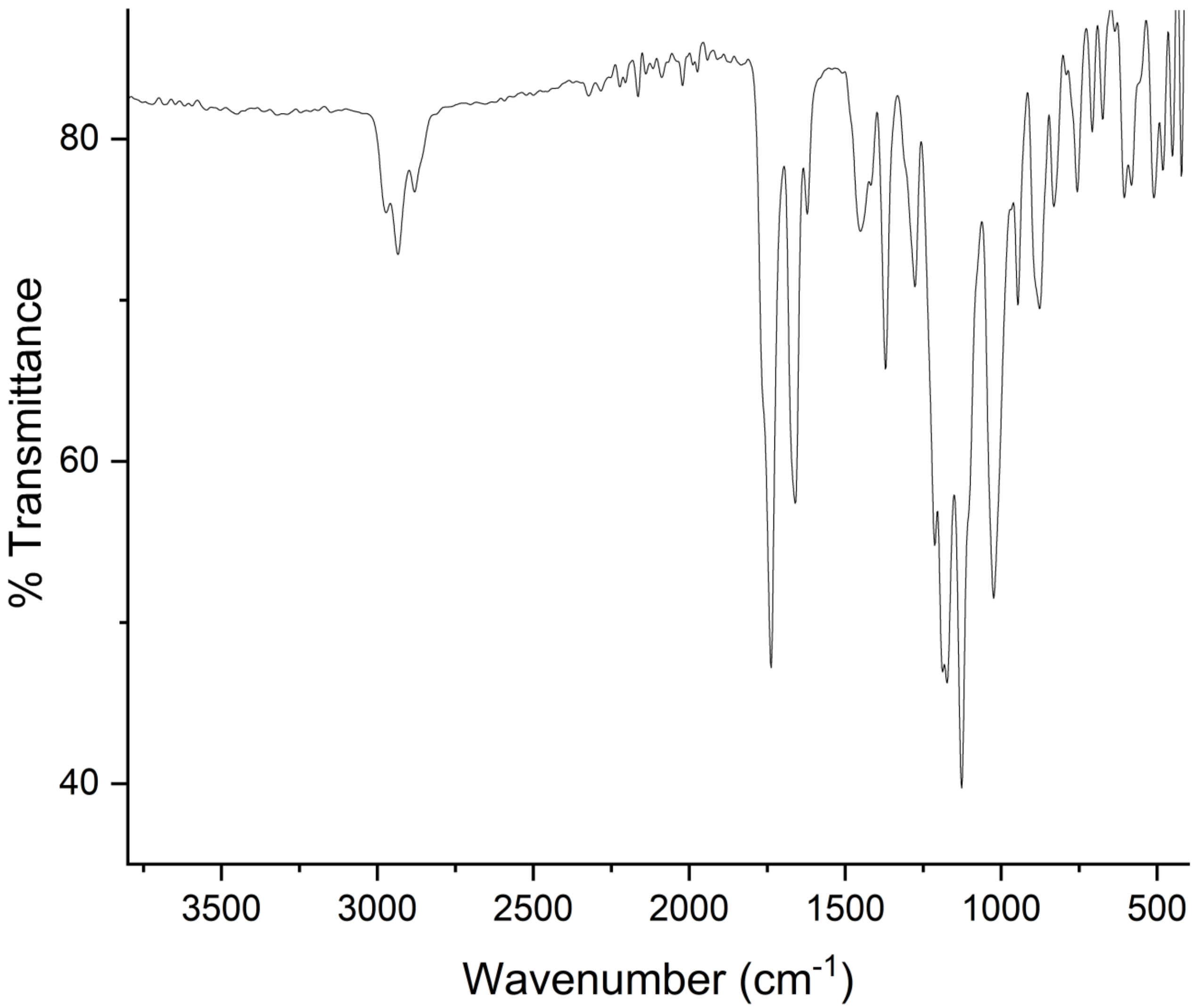

Figure S5.- FTIR spectrum of acetylated derivative (**1a**)

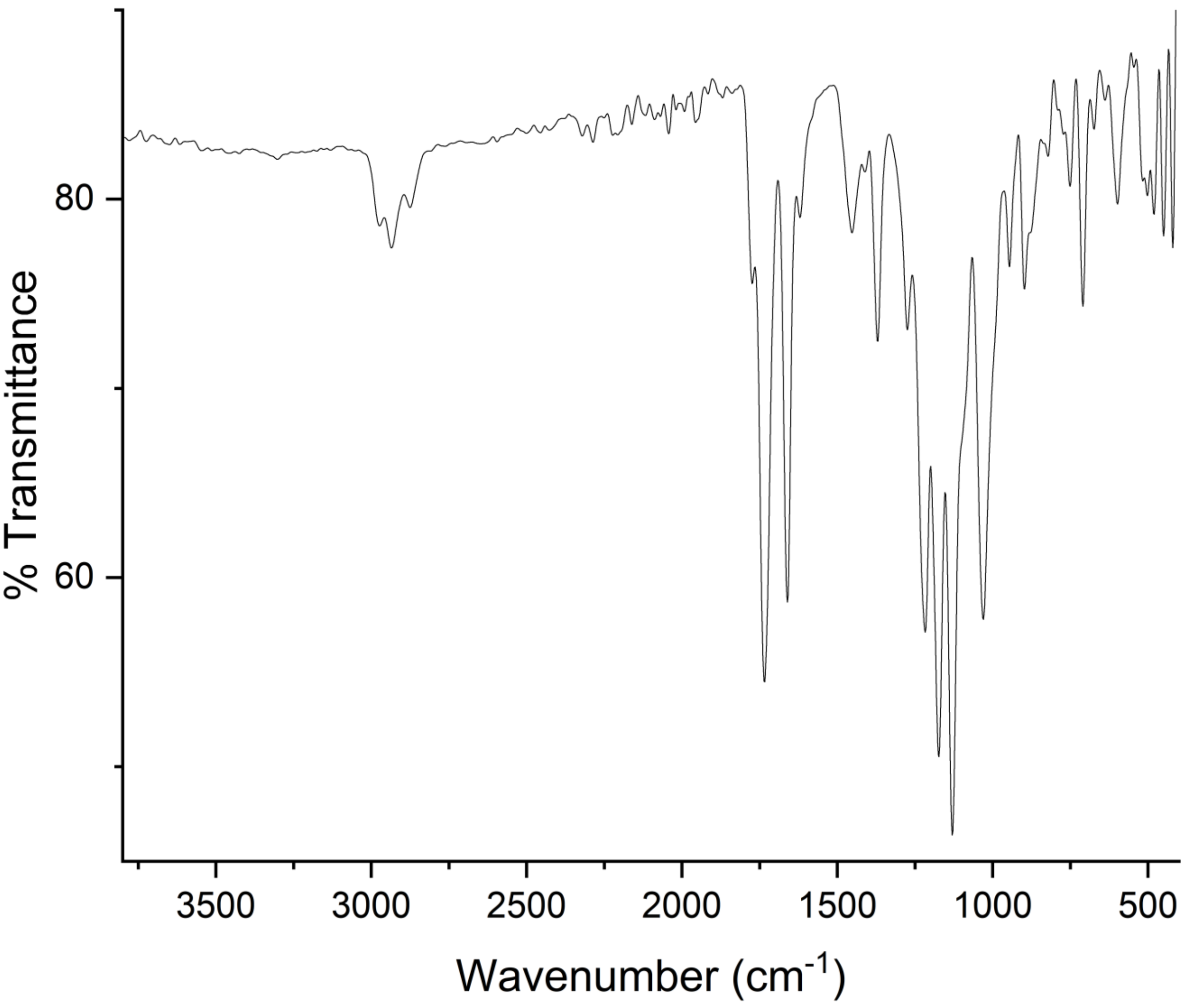

Figure S6.- FTIR spectrum of benzoylate derivative (**1b**)

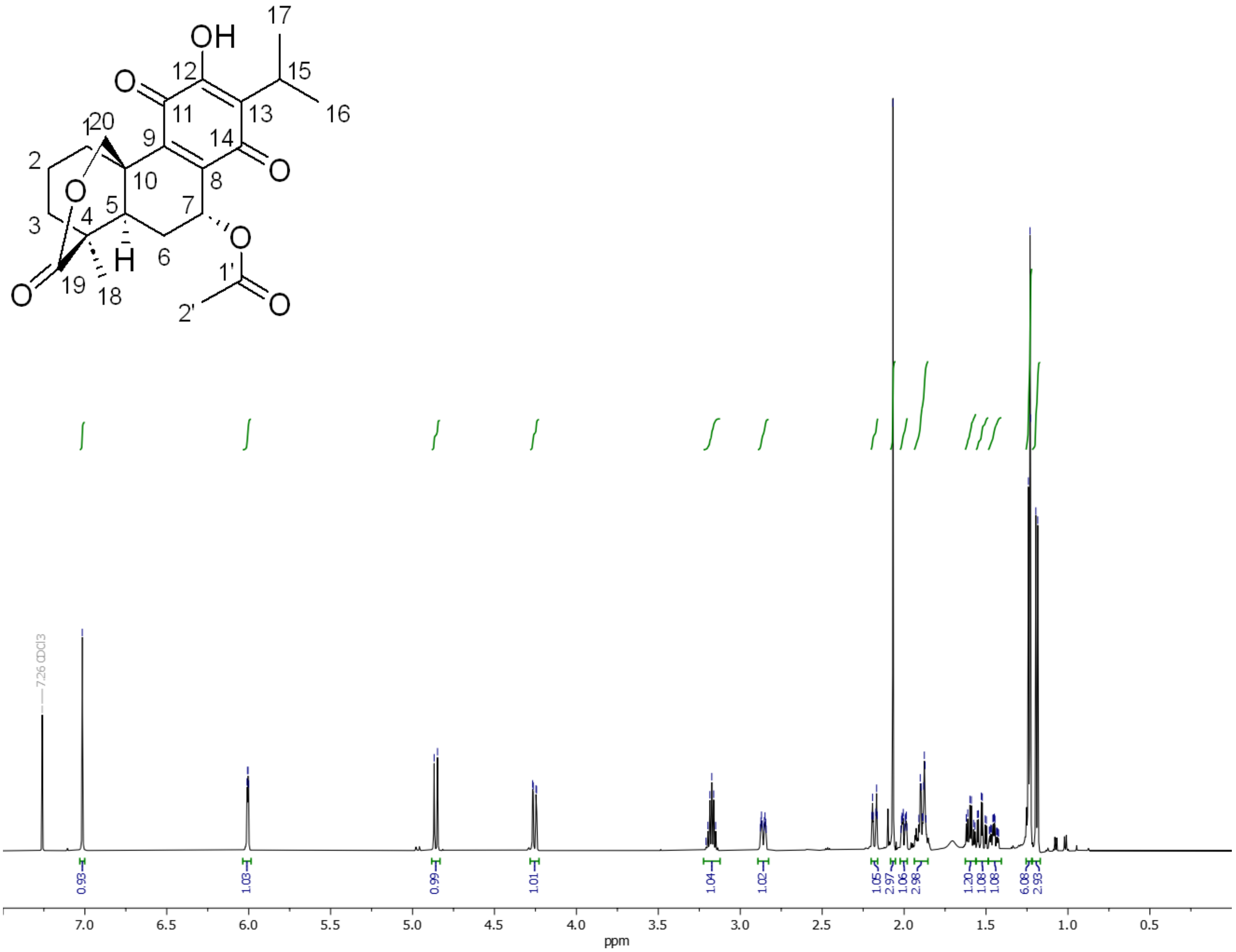

Figure S7.- ^{1}H spectrum of sessein (**1**)

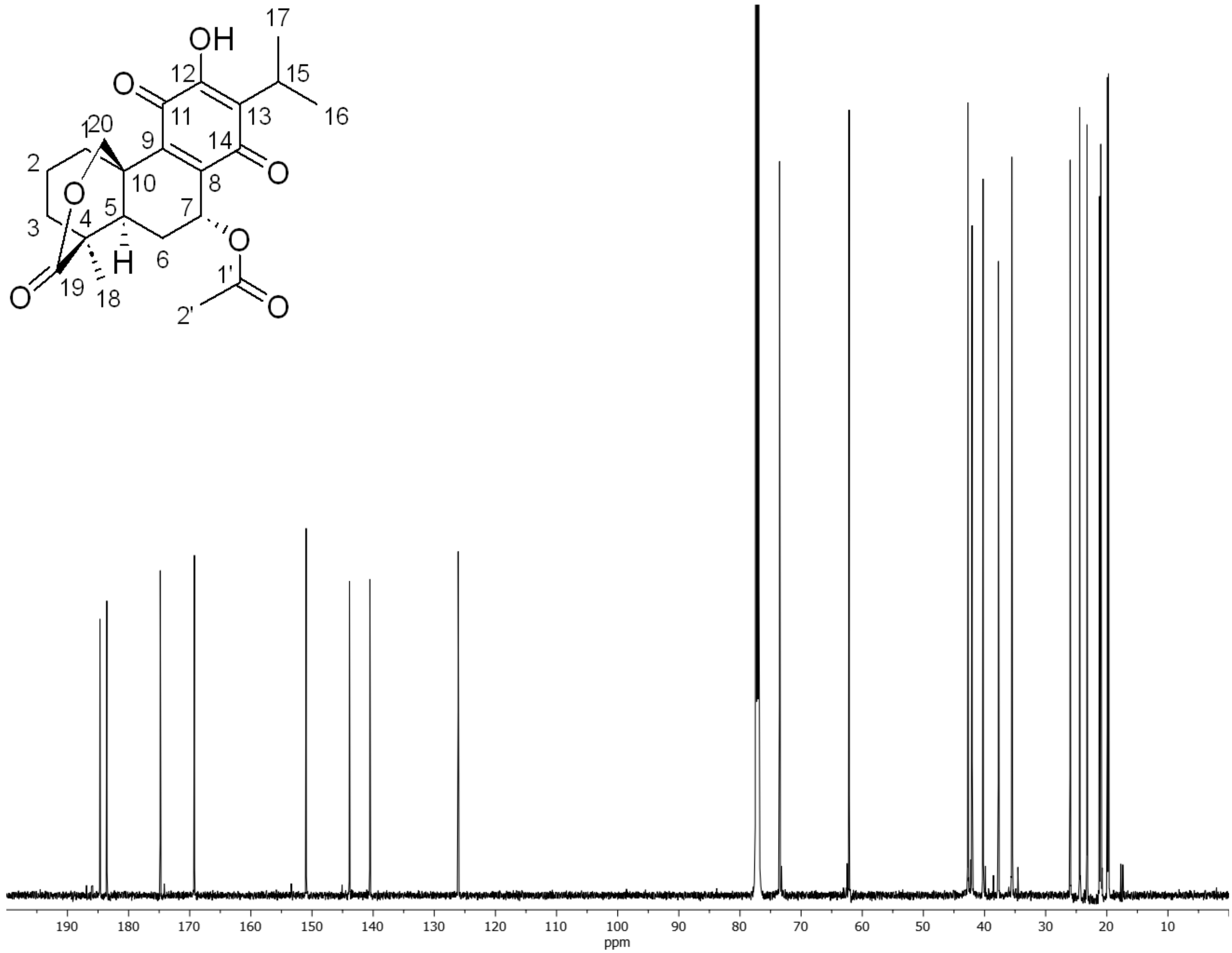

Figure S8.- ^{13}C spectrum of sessein (**1**)

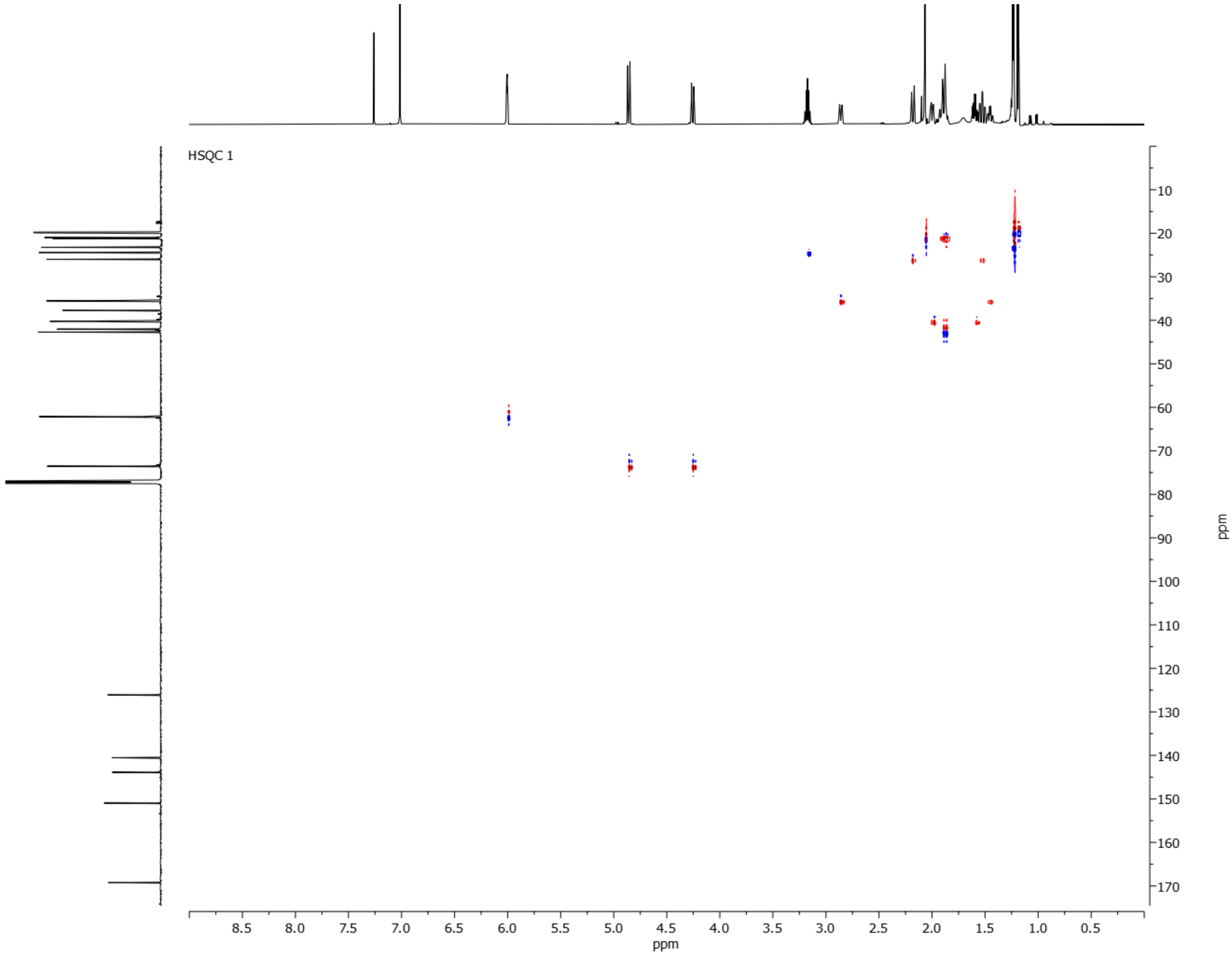

Figure S9.- HSQC spectrum of sessein (**1**)

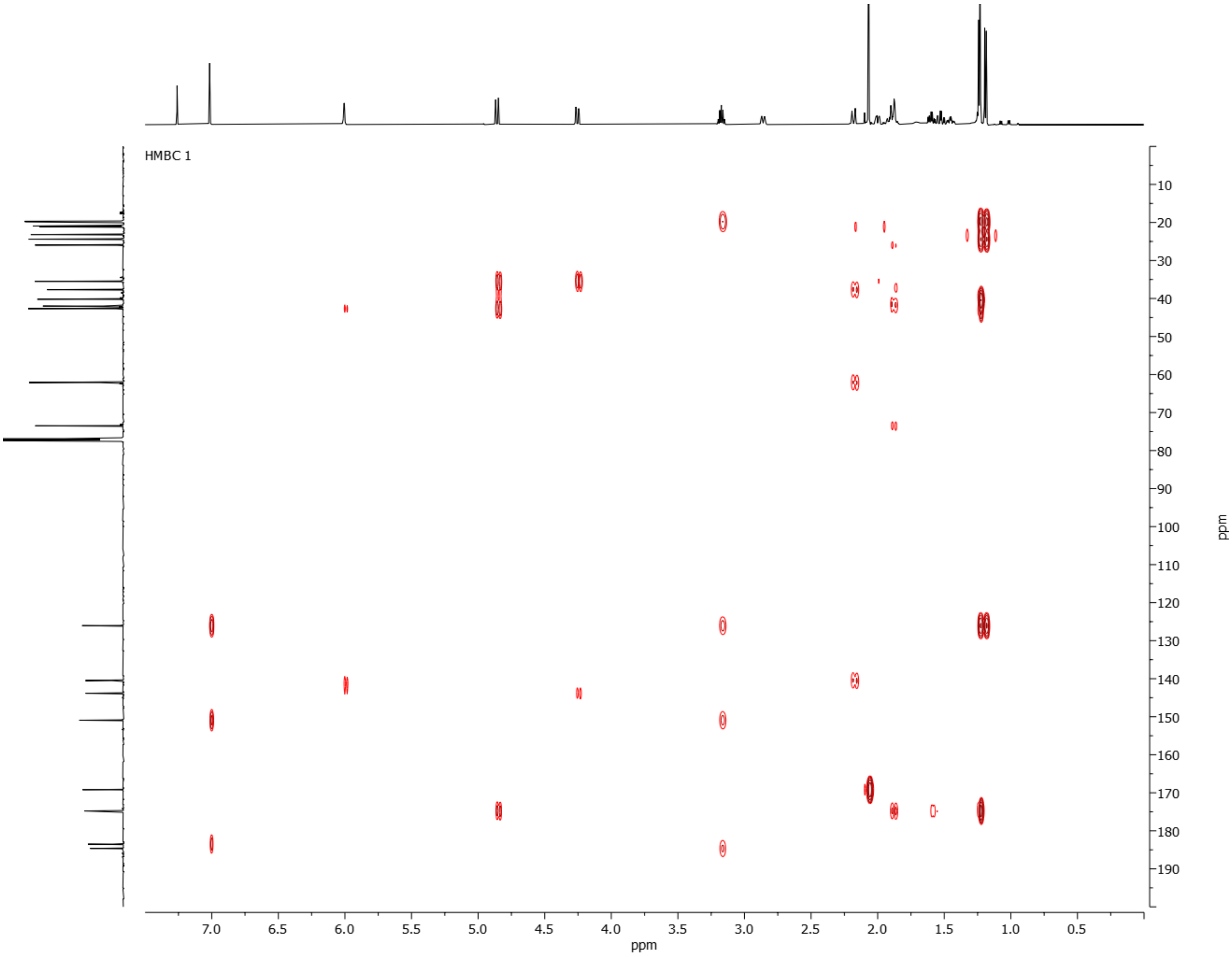

Figure S10.- HMBC spectrum of sessein (**1**)

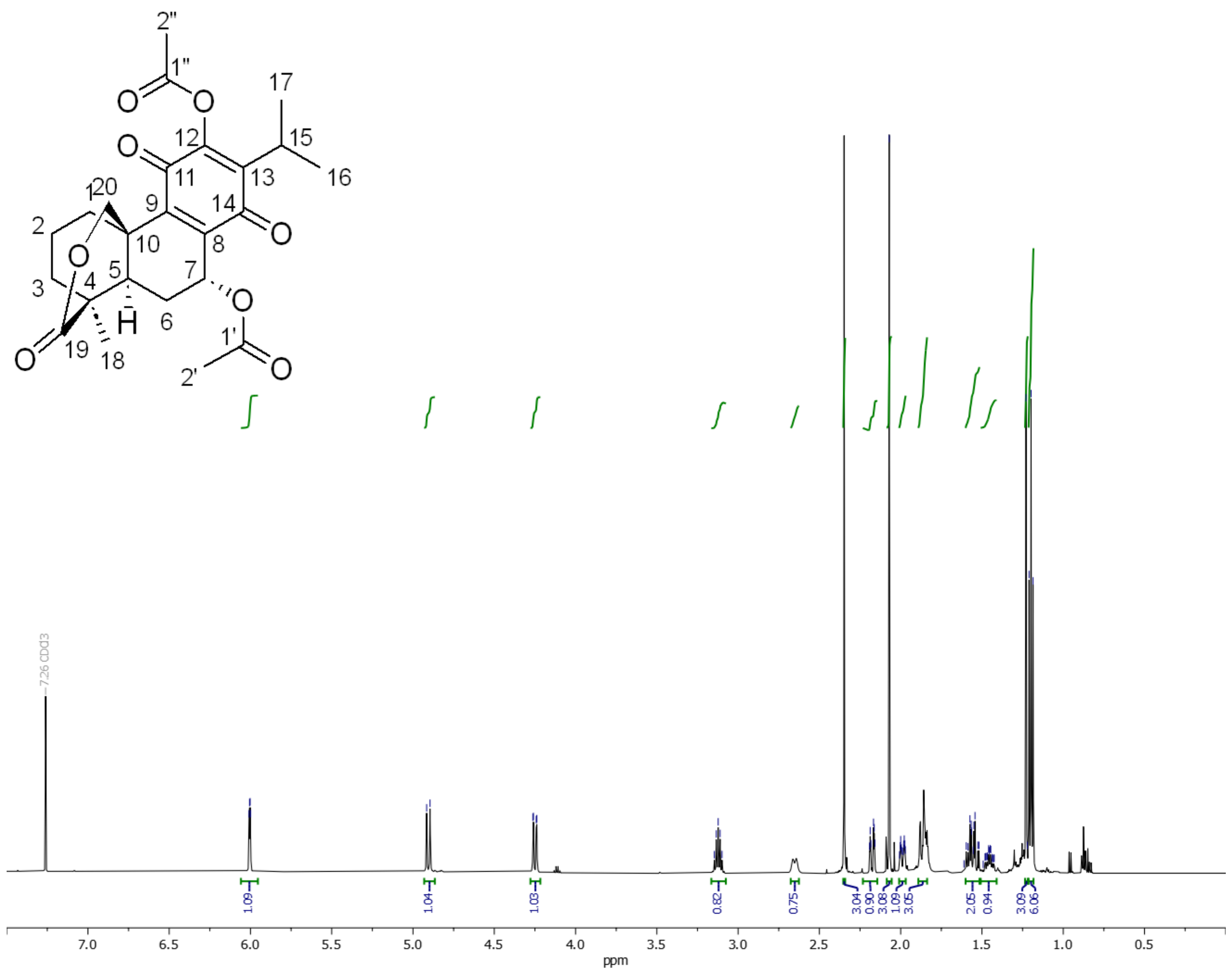

Figure S11.- ¹H spectrum of acetylated derivative (**1a**)

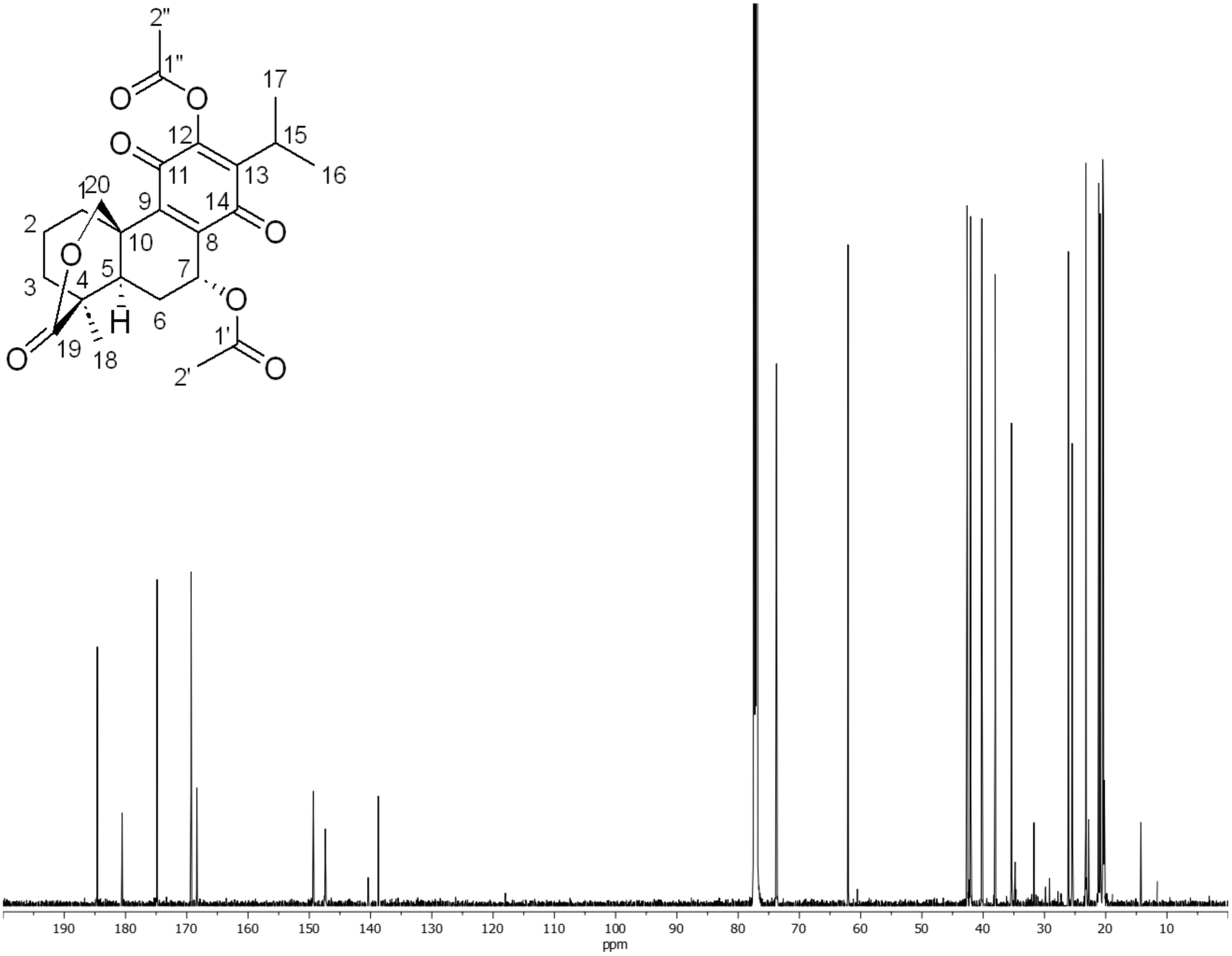

Figure 12.- ^{13}C spectrum of acetylated derivative (**1a**)

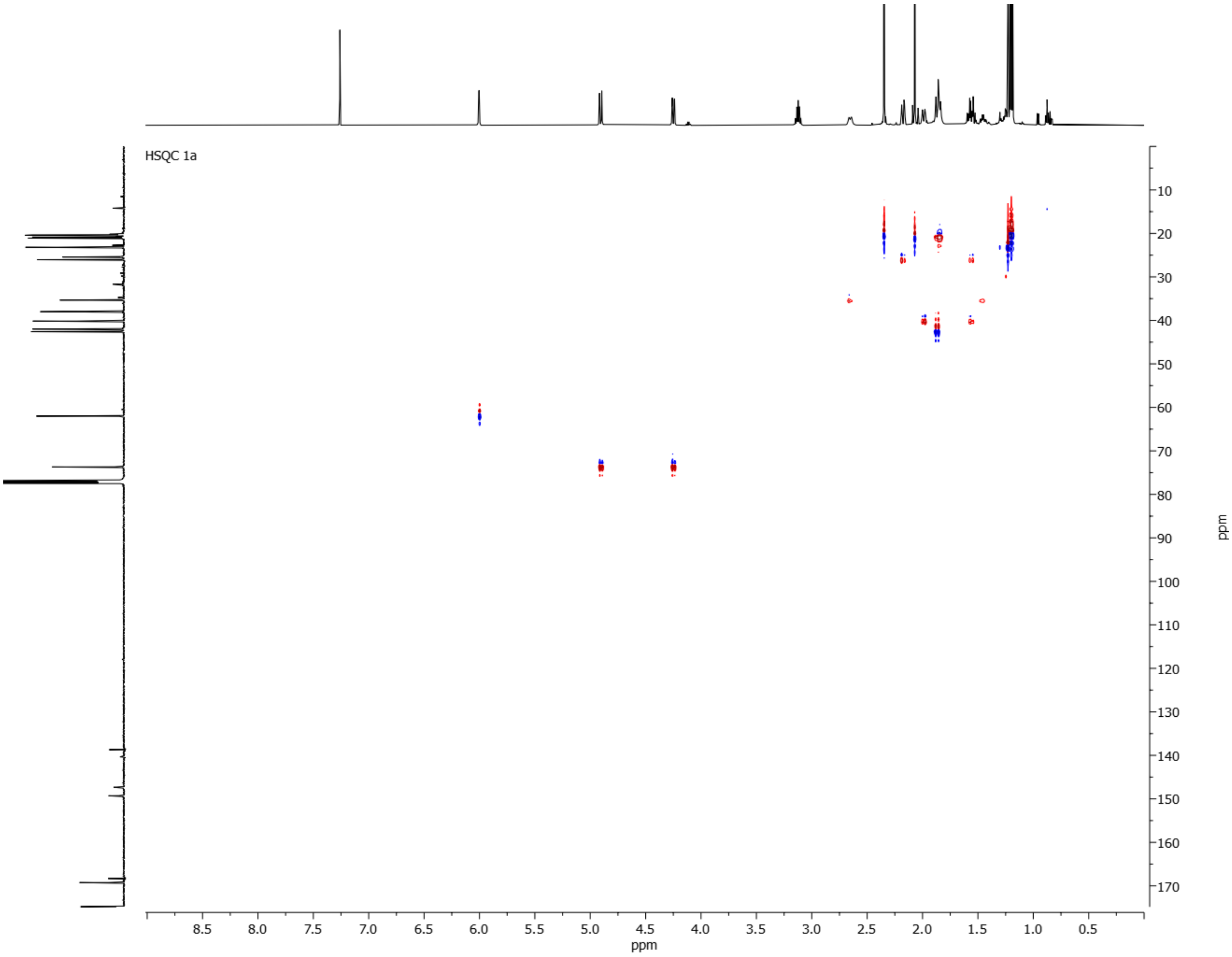

Figure S13.- HSQC spectrum of acetylated derivative (**1a**)

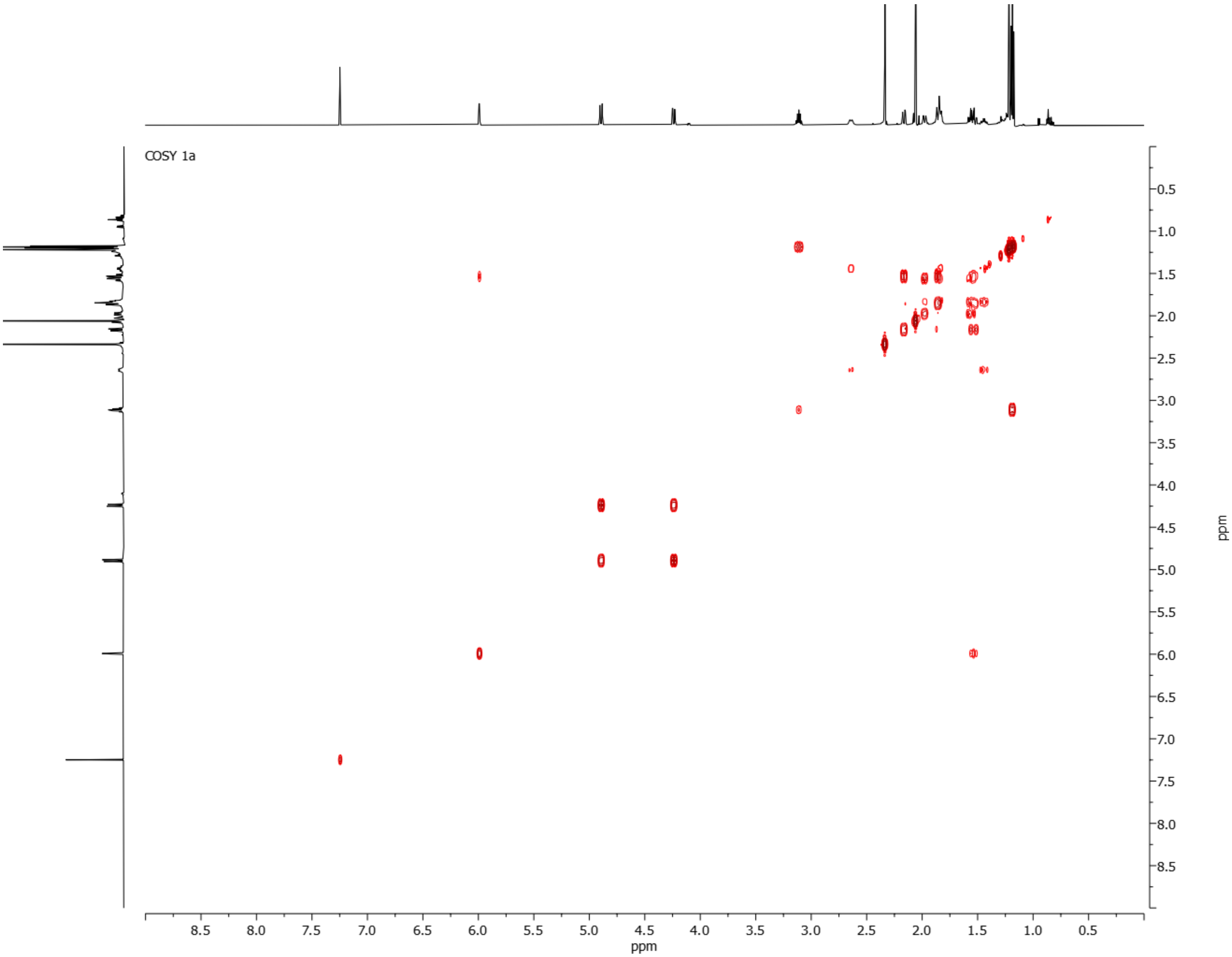

Figure S14.- COSY spectrum of acetylated derivative (**1a**)

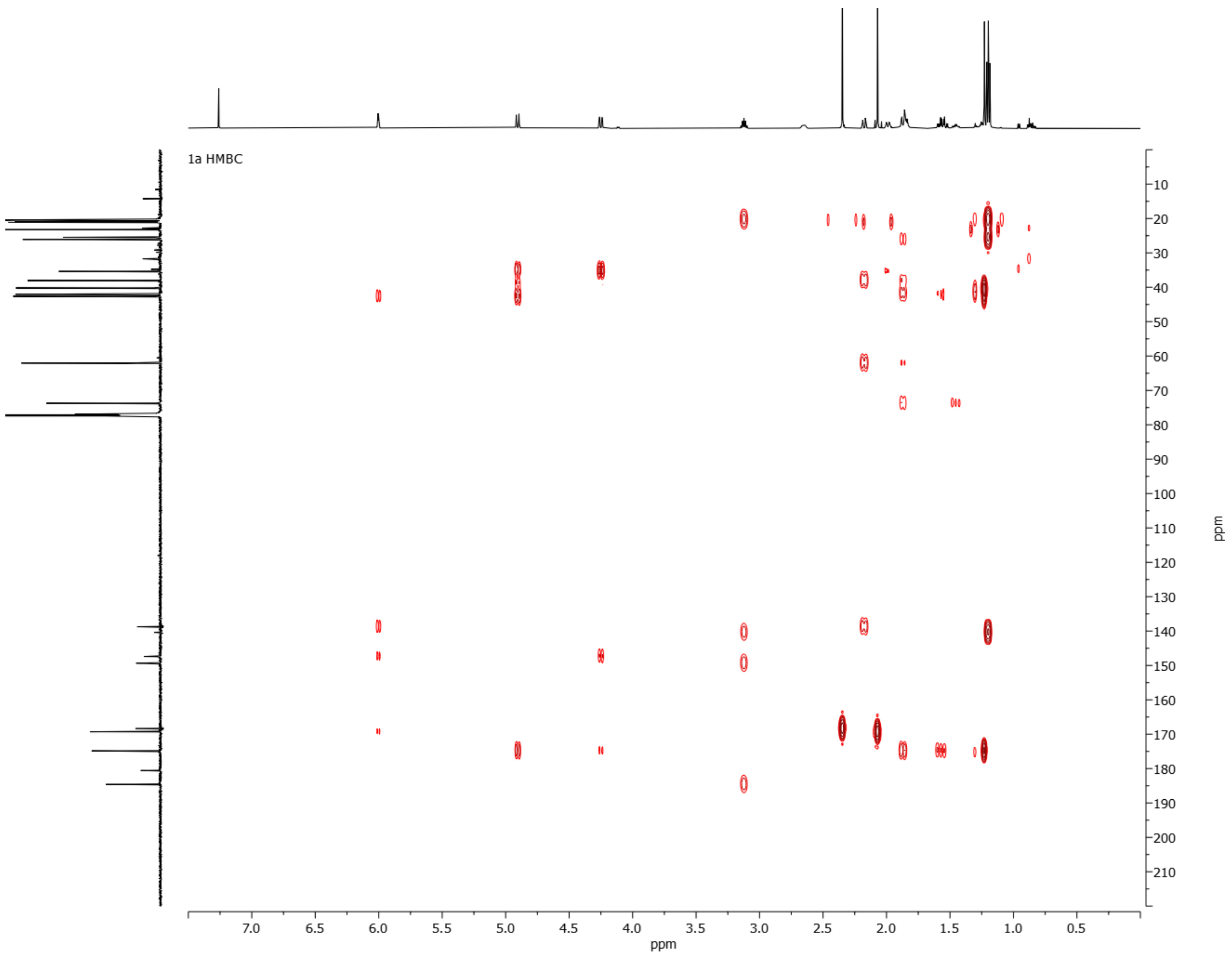

Figure S15.- HMBC spectrum of acetylated derivative (**1a**)

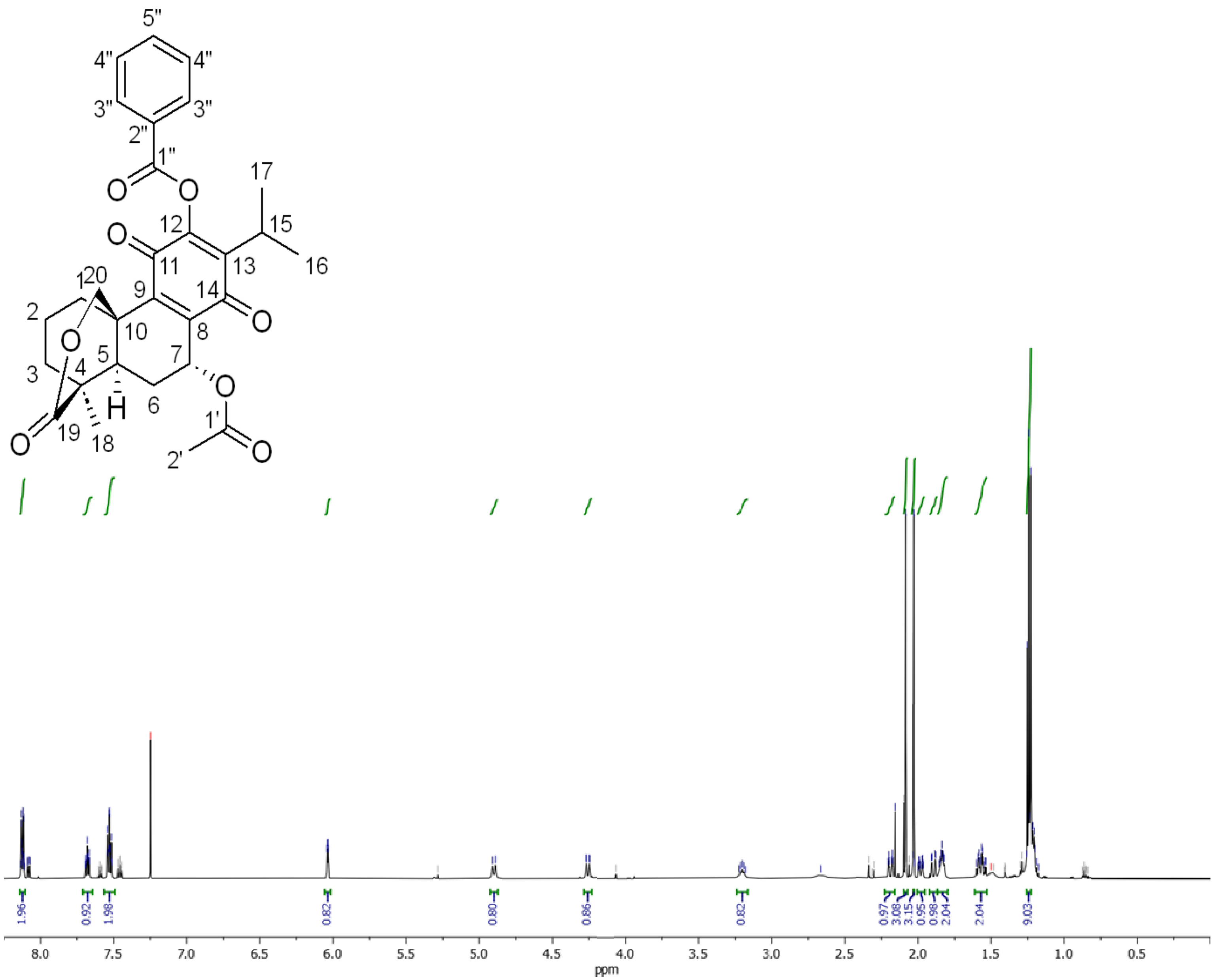

Figure S16.- ^{1}H spectrum of benzoylate derivative (**1b**)

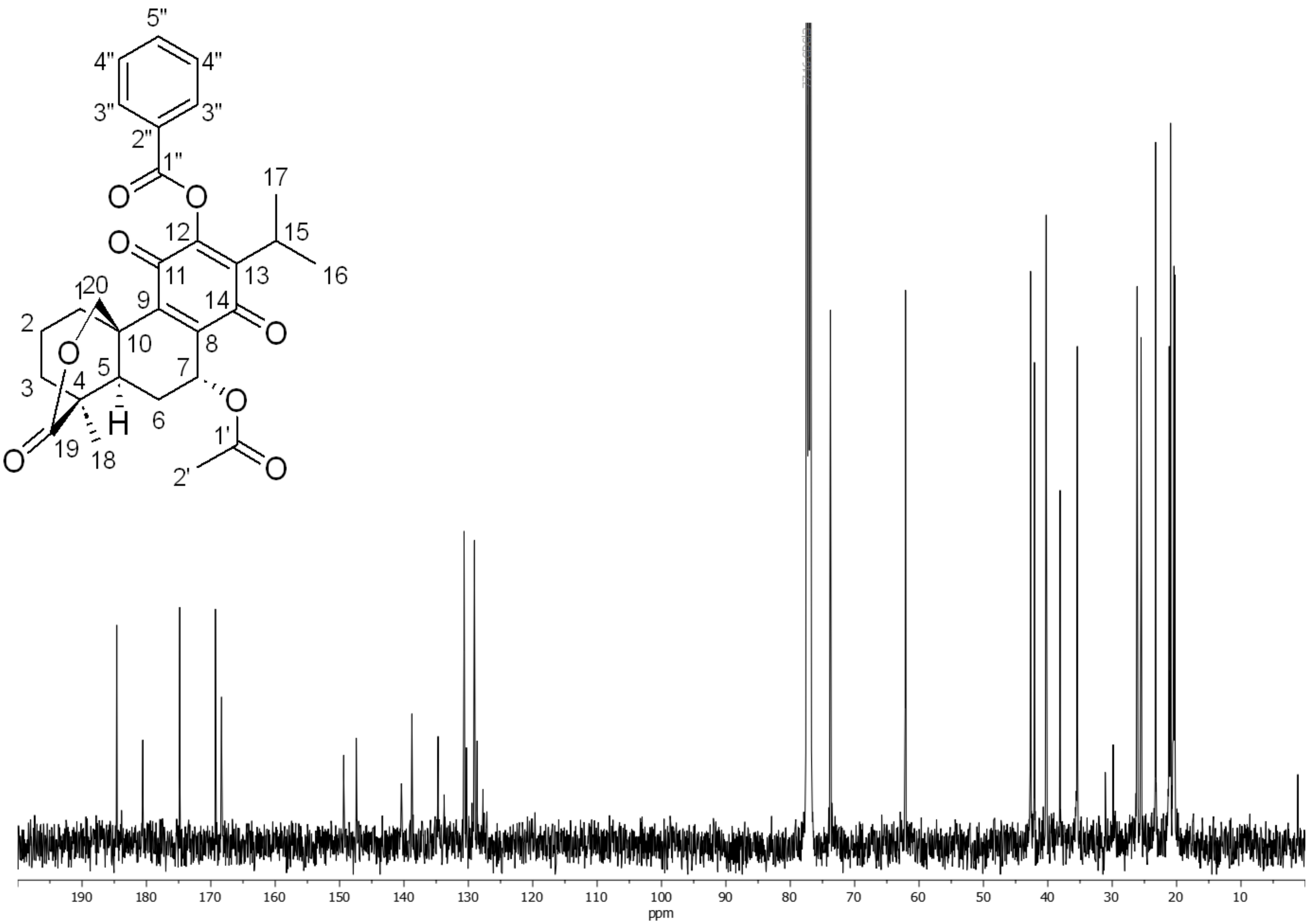

Figure S17.- ^{13}C spectrum of benzoylate derivative (**1b**)

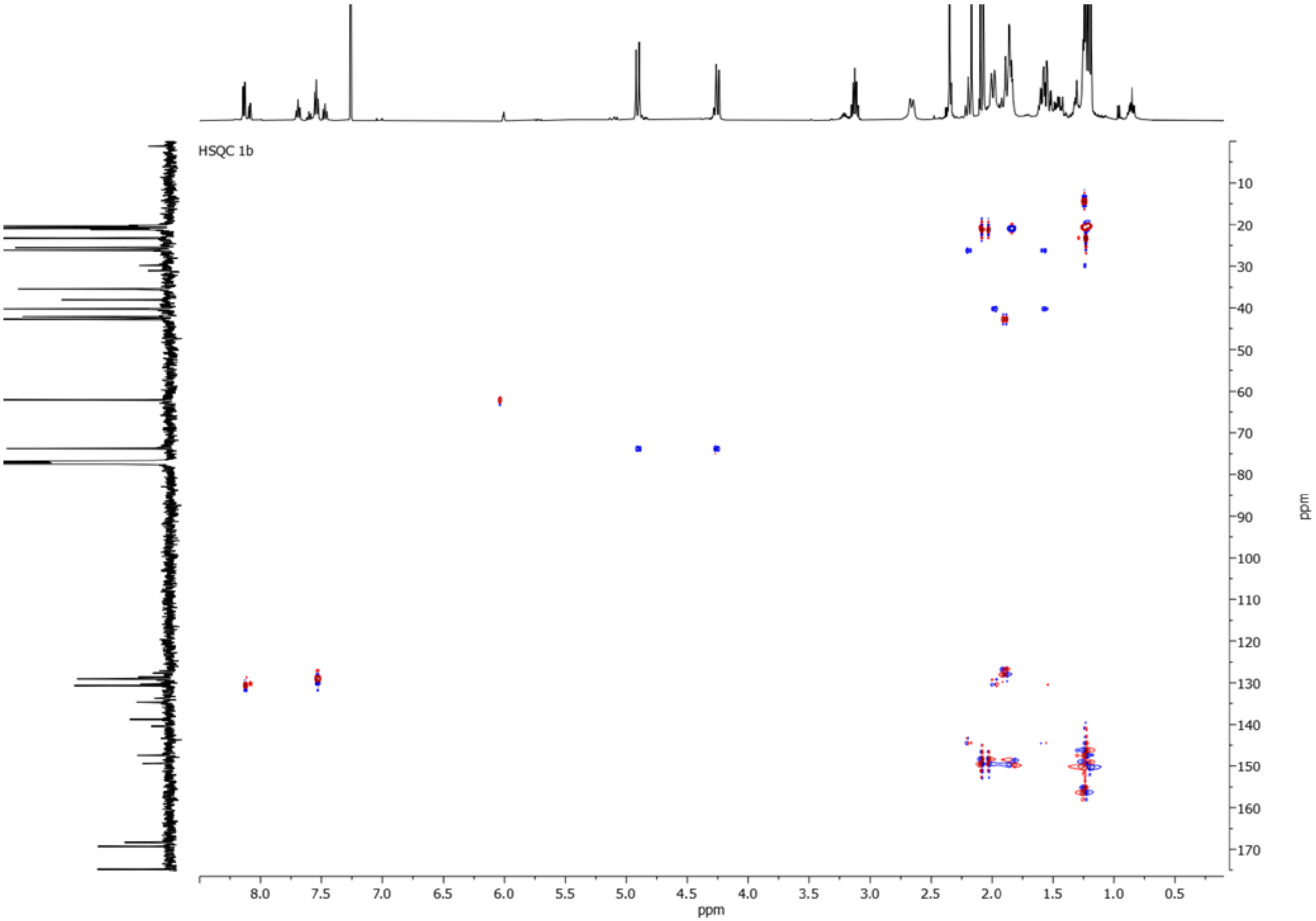

Figure S18.- HSQC spectrum of benzoylate derivative (**1b**)

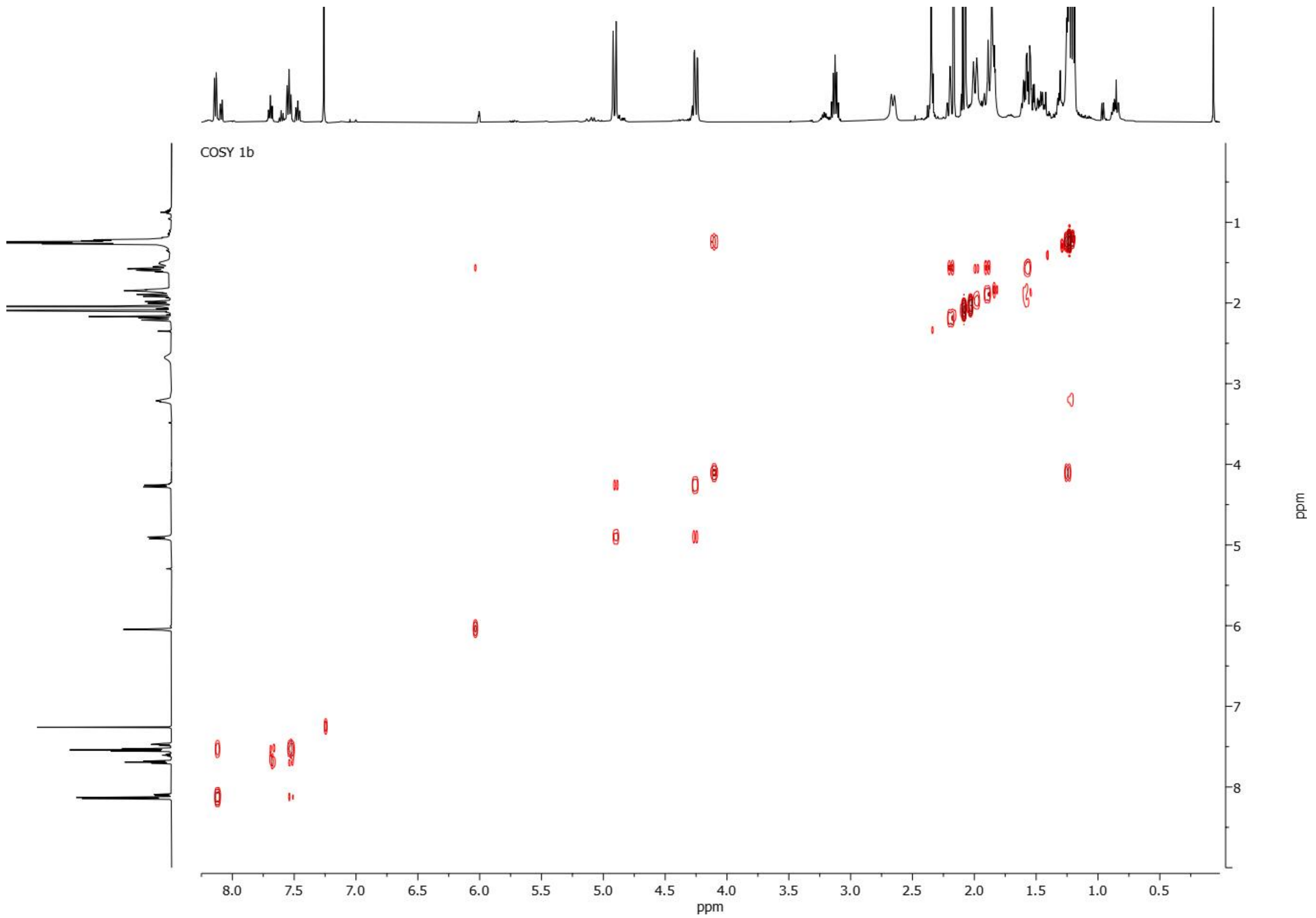

Figure S19.- COSY spectrum of benzoylate derivative (**1b**)

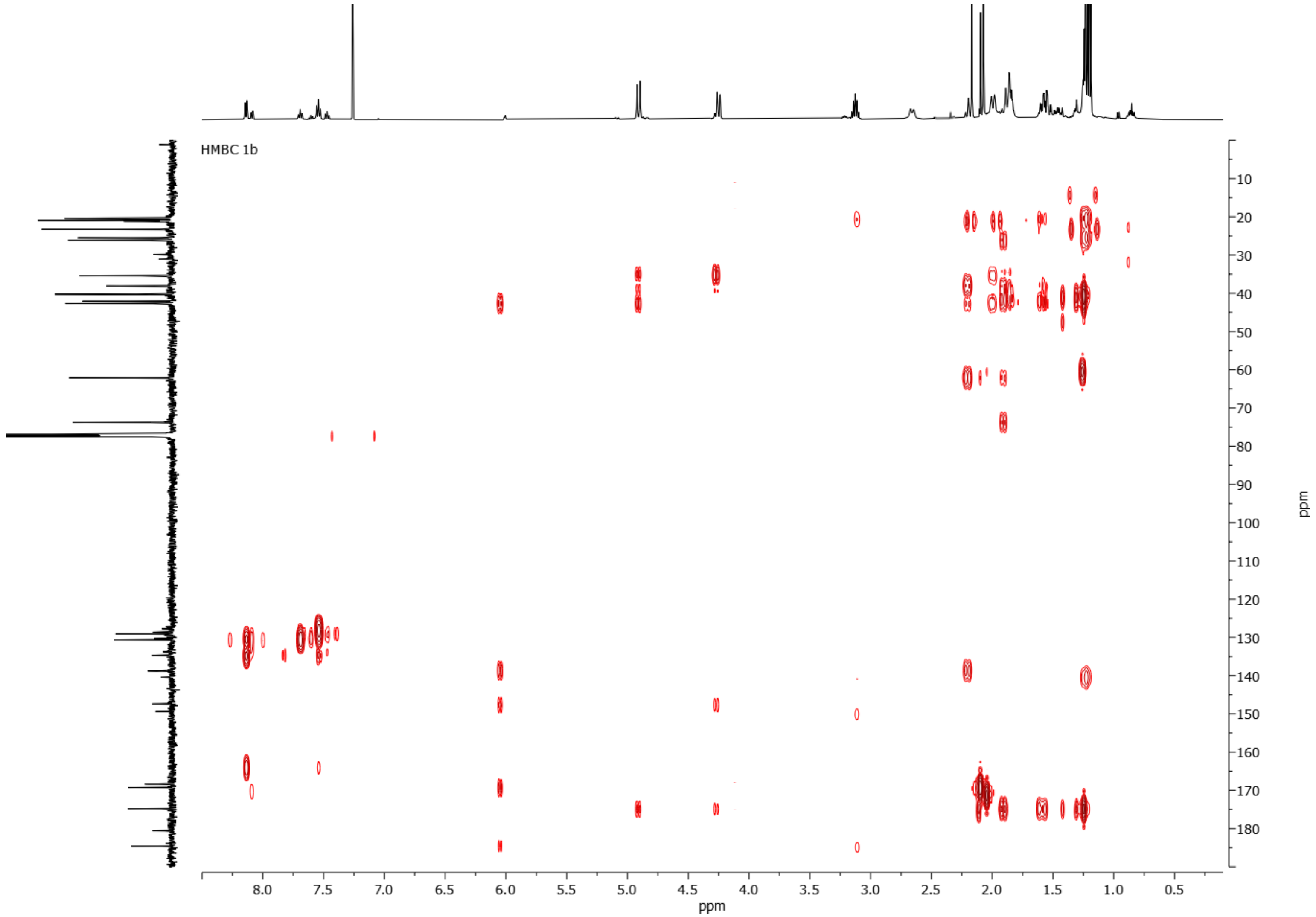

Figure S20.- HMBC spectrum of benzoylate derivative (**1b**)
